# White matter hyperintensities precede other biomarkers in *GRN* frontotemporal dementia

**DOI:** 10.1002/alz.70695

**Published:** 2025-10-07

**Authors:** Mahdie Soltaninejad, Mahsa Dadar, D. Louis Collins, Reza Rajabli, Vikram Venkatraghavan, Arabella Bouzigues, Lucy L. Russell, Phoebe H. Foster, Eve Ferry‐Bolder, John C. van Swieten, Lize C. Jiskoot, Harro Seelaar, Raquel Sanchez‐Valle, Robert Laforce, Caroline Graff, Daniela Galimberti, Rik Vandenberghe, Alexandre de Mendonça, Pietro Tiraboschi, Isabel Santana, Alexander Gerhard, Johannes Levin, Benedetta Nacmias, Markus Otto, Maxime Bertoux, Thibaud Lebouvier, Chris R. Butler, Isabelle Le Ber, Elizabeth Finger, Maria Carmela Tartaglia, Mario Masellis, James B. Rowe, Matthis Synofzik, Fermin Moreno, Barbara Borroni, Jonathan D. Rohrer, Yasser Iturria‐Medina, Simon Ducharme, GENFI Consortium

**Affiliations:** ^1^ McConnell Brain Imaging Centre Montreal Neurological Institute McGill University Montréal Québec Canada; ^2^ Douglas Mental Health University Institute Department of Psychiatry McGill University Montréal Québec Canada; ^3^ Department of Biomedical Engineering and Physics Amsterdam University Medical Center University of Amsterdam Amsterdam the Netherlands; ^4^ Informatics Institute University of Amsterdam Amsterdam the Netherlands; ^5^ Dementia Research Centre Department of Neurodegenerative Disease UCL Queen Square Institute of Neurology London UK; ^6^ Department of Neurology Erasmus Medical Centre Rotterdam Netherlands; ^7^ Alzheimer's disease and Other Cognitive Disorders Unit, Neurology Service, Hospital Clínic Institut d'Investigacións Biomèdiques August Pi I Sunyer University of Barcelona Barcelona Spain; ^8^ Clinique Interdisciplinaire de Mémoire Département des Sciences Neurologiques CHU de Québec, and Faculté de Médecine Université Laval Québec Canada; ^9^ Department of Neurobiology Care Sciences and Society Center for Alzheimer Research Division of Neurogeriatrics Bioclinicum Karolinska Institutet Solna Sweden; ^10^ Fondazione Ca’ Granda IRCCS Ospedale Policlinico Milano Italy; ^11^ Department of Biomedical Surgical and Dental Sciences University of Milan Milan Italy; ^12^ Laboratory for Cognitive Neurology Department of Neurosciences KU Leuven Leuven Belgium; ^13^ Faculty of Medicine University of Lisbon Lisbon Portugal; ^14^ Fondazione IRCCS Istituto Neurologico Carlo Besta Milano Italy; ^15^ University Hospital of Coimbra (HUC) Neurology Service Faculty of Medicine University of Coimbra Coimbra Portugal; ^16^ Division of Psychology Communication and Human Neuroscience Wolfson Molecular Imaging Centre University of Manchester Manchester UK; ^17^ Department of Nuclear Medicine Center for Translational Neuro‐ and Behavioral Sciences University Hospital Essen Essen Germany; ^18^ Department of Geriatric Medicine Arnsberg Germany; ^19^ Department of Neurology LMU University Hospital Munich Germany; ^20^ Germany Center for Neurodegenerative Diseases Munich Germany; ^21^ Munich Cluster for Systems Neurology (SyNergy) Munich Germany; ^22^ Department of Neuroscience Psychology Drug Research and Child Health University of Florence Florence Italy; ^23^ IRCCS Fondazione Don Carlo Gnocchi Florence Italy; ^24^ Department of Neurology University of Ulm Ulm Germany; ^25^ Centre Hospitalier Universitaire de Lille, Nord département Lille France; ^26^ Nuffield Department of Clinical Neurosciences, Medical Sciences Division University of Oxford Oxford UK; ^27^ Sorbonne Université Paris Brain Institute – Institut du Cerveau – ICM Hôpital Pitié Paris France; ^28^ Department of Clinical Neurological Sciences London Health Sciences Centre University Hospital University of Western Ontario London Ontario Canada; ^29^ Tanz Centre for Research in Neurodegenerative Diseases University of Toronto Toronto Ontario Canada; ^30^ Sunnybrook Health Sciences Centre Sunnybrook Research Institute University of Toronto Toronto Ontario Canada; ^31^ Department of Clinical Neurosciences Cambridge University Hospitals NHS Trust, and MRC Cognition and Brain Sciences Unit University of Cambridge Cambridge UK; ^32^ Department of Neurodegenerative Diseases Hertie‐Institute for Clinical Brain Research and Center of Neurology, University of Tübingen Tübingen Germany; ^33^ Cognitive Disorders Unit Department of Neurology Donostia Universitary Hospital Donostia Gipuzkoa Spain; ^34^ Department of Clinical and Experimental Sciences University of Brescia Brescia Italy; ^35^ Molecular Markers Laboratory IRCCS Istituto Centro San Giovanni di Di Brescia Italy

**Keywords:** biomarker sequence, C9orf72, dementia, disease progression, early marker, event‐based modeling, FTD, GRN, MAPT, magnetic resonance imaging, neurodegeneration, neurofilament light chain, neuroimaging, progranulin, white matter

## Abstract

**INTRODUCTION:**

Increased white matter hyperintensities (WMHs) have been reported in genetic frontotemporal dementia (FTD) in small studies, but the sequence of WMH abnormalities relative to other biomarkers is unclear.

**METHODS:**

Using a large dataset (*n* = 763 GENFI2 participants), we measured WMHs and examined them across genetic FTD variants and stages. Cortical and subcortical volumes were parcellated, and serum neurofilament light chain (NfL) levels were measured. Biomarker progression was assessed with discriminative event‐based and regression modeling.

**RESULTS:**

Symptomatic *GRN* carriers showed elevated WMHs, primarily in the frontal lobe, while no significant increase was observed in symptomatic *C9orf72* or *MAPT* carriers. WMH abnormalities preceded NfL elevation, ventricular enlargement, and cortical atrophy. Longitudinally, baseline WMHs predicted subcortical changes, while subcortical volumes did not predict WMH changes, suggesting WMHs may precede neurodegeneration.

**DISCUSSION:**

WMHs are elevated in a subset of *GRN*‐associated FTD. When present, they appear early and should be considered in disease progression models.

**Highlights:**

- Elevated WMH volumes are found predominantly in symptomatic *GRN*.
- WMH accumulation is mostly observed in the frontal lobe.
- WMH abnormalities appear early in *GRN*‐associated FTD, before NfL, atrophy, and ventriculomegaly.
- Longitudinally, WMH volumes can predict subcortical changes, but not vice versa.
- WMHs are key early markers in *GRN*‐associated FTD and should be included in progression models.

## 1 BACKGROUND

Frontotemporal dementia (FTD) presents as a multifaceted neurodegenerative disorder marked by progressive deterioration in behavior, personality, and/or language, ranking as the second most prevalent cause of early onset dementia following Alzheimer's disease.^1^ Approximately 30% of FTD cases exhibit a robust familial dementia history, often linked to specific genetic mutations. The majority of FTD's heritability stems from autosomal dominant mutations within three primary genes: *C9orf72* (chromosome 9 open reading frame 72), *GRN* (progranulin), and *MAPT* (microtubule‐associated protein tau).^2^ Despite symptomatic overlap across these gene mutations, the molecular mechanisms driving the emergence of phenotypic outcomes are inherently distinct.

White matter hyperintensities (WMHs) have garnered significant attention due to their clinicopathologic contributions in a range of neurodegenerative conditions and their deleterious effect on cognition.^3^, ^4^ These lesions manifest as hyperintensities on specific sequences of magnetic resonance imaging (MRI), signaling abnormalities within the brain's white matter. These anomalies can indicate areas of demyelination, gliosis, and/or small vessel disease. WMH *post mortem* histopathology reveals non‐specific brain alterations, such as gliosis, myelin and axon loss attributed to arteriosclerosis, tissue rarefaction, and lipohyalinosis. These changes may be caused by various factors, including hypoxia, hypoperfusion, blood–brain barrier leakage, inflammation, degeneration, and amyloid angiopathy.^5^ Moreover, increasing evidence suggests that WMHs in neurodegeneration are not solely driven by vascular pathology but may also reflect intrinsic disease processes, including amyloidosis and gray matter degeneration, as shown in Alzheimer's disease, where WMHs have been linked to cerebral amyloid angiopathy and neurodegeneration rather than traditional vascular risk factors.^6^

The overwhelming majority of neuroimaging research in FTD has centered on changes in gray matter, with less attention given to the role of WMHs. Sudre et al.^7^ reported increased WMH burden in FTD patients with symptomatic *GRN* mutations, but not in those carrying *MAPT* or *C9orf72* mutations. This investigation did not find significant WMH changes during the presymptomatic phase. A follow‐up longitudinal study from the same group showed variations among *GRN* cases, with 25% of individuals displaying either no WMHs or only mild WMHs during the symptomatic phase and only 9% of those in the presymptomatic phase already exhibiting severe WMH involvement.^8^ Despite its reliance on a small sample size, this study has exerted a significant influence on the research landscape concerning the association between FTD and WMHs being tied to *GRN* cases. Another study^9^ employing diffusion tensor imaging revealed microstructural white matter changes among individuals with *C9orf72* repeat expansions and *MAPT* mutations during presymptomatic stages, highlighting the impact that genetic FTD has on white matter.

Despite these results, WMHs were not factored into recent disease models aiming to guide clinical trials.^10^ In our study, we aimed to address these gaps in knowledge by investigating the prevalence of WMH across distinct genetic groups and different stages of FTD, leveraging a later version of the GENFI2 dataset with a larger sample to assess the validity of previous WMH studies. We further undertook a temporal analysis of WMH compared to other markers in the course of the progression of the disease.

## 2 METHODS

### 2.1 Data

Data for this study were obtained from the fifth data freeze of GENFI2 (Genetic Frontotemporal dementia Initiative), a large international study involving 25 centers in Europe and Canada. GENFI collects longitudinal data on genetic FTD and aims to gather multimodal neuroimaging, cognitive, and fluid biomarkers to develop markers for early‐stage FTD identification, track disease progression, and gain insight into the presymptomatic phase of the disease.

Participants included in GENFI2 were known symptomatic carriers of pathogenic mutations in *C9orf72*, *GRN*, or *MAPT*, as well as their first‐degree relatives who were at risk of carrying a mutation. Genotyping was performed at local sites, and all participants underwent a standard clinical evaluation, including medical and family history assessments, as well as physical examinations. Symptomatic carriers met clinical criteria for behavioral variant of frontotemporal dementia (bvFTD), primary progressive aphasia (PPA), frontotemporal dementia with amyotrophic lateral sclerosis (FTD‐ALS) or other rare presentations, while presymptomatic carriers did not fulfill clinical criteria. The non‐carrier group comprised healthy first‐degree relatives of symptomatic carriers who tested negative for the reported family mutation. Detailed inclusion and exclusion criteria can be found elsewhere.^11^

T1‐weighted and T2‐weighted MRI scans were acquired across multiple sites using 3T scanners from Siemens (Trio, Skyra, Prisma), Philips, and General Electric. Imaging followed harmonized acquisition protocols established by the GENFI study to ensure inter‐site consistency.^11^ For T1‐weighted imaging, sagittal 3D MPRAGE or equivalent sequences were used. Acquisition parameters (median [range]) included an inversion time (TI) of 850 ms (400 to 960 ms), repetition time (TR) of 2000 ms (6.6 to 2200 ms), echo time (TE) of 2.9 ms (2.2 to 9.0 ms), flip angle of 8° (8° to 11°), slice thickness of 1.1 mm, and 208 slices (200 to 208). For T2‐weighted imaging, sagittal 3D fast spin echo sequences were used. Acquisition parameters (median [range]) included TR of 3200 ms (2200 to 3200 ms), effective TE (TEeff) of 105 ms (50 to 105 ms), slice thickness of 1.1 mm, and 176 slices (176 to 208).

Serum levels of neurofilament light chain (NfL) and glial fibrillary acidic protein (GFAP) were longitudinally measured using the single‐molecule array technique (Simoa).^12^

**RESEARCH IN CONTEXT**

1. **Systematic review**: We systematically reviewed the literature on WMHs in FTD using PubMed. While a few small studies reported increased WMHs in *GRN* mutation carriers, their sample sizes were limited, and they did not assess the timing of WMHs within disease progression or their temporal relationship to other biomarkers.
2. **Interpretation**: We identified a sequence of key biomarkers in *GRN*‐associated FTD and demonstrated that WMHs were among the earliest biomarkers, preceding cortical and subcortical atrophy as well as blood biomarkers. This aligns with neuropathological evidence of early white matter involvement in FTLD‐*GRN*. Additionally, using a larger dataset, we validated previous reports of elevated WMHs in *GRN* carriers, confirming their reliability.
3. **Future directions**: Future studies should integrate WMHs into FTD progression models to enhance early diagnosis. Understanding why only a subset of *GRN* carriers exhibit high WMH volumes remains a key research priority.

GENFI is a longitudinal dataset with multiple visits available for some participants. In this study, we first used a cross‐sectional design to maximize the number of subjects, selecting only one visit per participant (except in our final analysis, which incorporated a longitudinal approach). To ensure consistency in data selection, we prioritized visits with both blood and imaging biomarkers available. Where participants had multiple eligible visits, we chose the most recent one to better capture a broader spectrum of disease severity. Longitudinal visits were used to test for the temporal relationship of biomarker changes.

### 2.2 Image processing

#### 2.2.1 White matter hyperintensities segmentation

**FIGURE 1:**
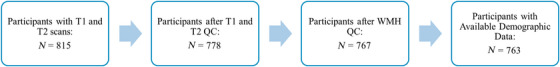
Flowchart of inclusion and exclusion criteria in study. QC, quality control; WMH, white matter hyperintensity.

The segmentation of WMH was performed using BISON,^13^ integrating data from T1‐weighted (T1w) and T2‐weighted (T2w) imaging modalities. The workflow employed a random forest classifier trained with location, intensity parameters, and manually labeled data to generate participant‐specific WMH maps. Before WMH segmentation, T1w and T2w scans underwent preprocessing steps including image denoising, intensity non‐uniformity correction, and intensity normalization within a range of 0‐100. T1w images were linearly registered and subsequently nonlinearly registered to the ICBM152 template. T1w and T2w images were linearly co‐registered using a six‐parameter rigid registration. All stereotaxic space priors and averages for WMH segmentation were resampled onto the native T1w volume using the inverse of the estimated non‐linear registration transformation. Quality control was performed via visual inspection of WMH segmentations and corresponding structural MRI scans using Qrater.^14^ Eleven of the 778 participants were excluded due to segmentation errors or major image artifacts. This included cases in which visible hyperintensities were not captured by the segmentation, as well as cases with substantial over‐segmentation (Figure 1). The WMH lesion maps were then linearly registered to the ICBM152 template, and Hammers' atlas was used to quantify WMH volumes in each of the eight lobes.^15^, ^16^

#### 2.2.2 Brain parcellation

In this study, gray matter volumes were obtained with the Geodesic Information Flow (GIF) algorithm,^17^ a multi‐atlas segmentation approach, for accurate and robust cortical and subcortical volume parcellation. GIF utilizes spatially‐variant graph structures, connecting morphologically similar participants for gradual information diffusion amid large‐scale morphological variability. From the parcellated regions, we considered the volumes of cortical and subcortical areas most closely associated with FTD, as identified in previous studies,^18^, ^19^, ^20^, ^21^ including the frontal lobe, temporal lobe, insula, basal ganglia (nucleus accumbens, caudate, putamen, and globus pallidus), cerebellum, cingulate cortex, ventricle, amygdala, hippocampus, and thalamus. The summed volumes of left and right regions were then utilized for further analysis. Additionally, to address individual variations in brain size, the volumes were standardized by dividing them by the intracranial volume.

### 2.3 Statistical tests

Statistical analyses were conducted using RStudio Version 4.3.1. To achieve a normal distribution of the WMH volumes, a log transformation was applied. A linear regression model was employed to adjust the WMH volumes of mutation carriers, with age and sex being taken into account. The model was specified as follows:

(1) $$\log\left(\mathrm{WMH}\;\mathrm{volume}\right)∼\;\mathrm{Age}+\mathrm{Sex}\;$$

The model was fitted using data from the healthy control cohort. Subsequently, the difference between the actual log‐transformed WMH volumes and the predicted values derived from this model was calculated for each person. These differences, referred to as adjusted WMH values, represent the residual WMH volume after accounting for demographic variables.

During this stage of the analysis, four participants were excluded due to missing demographic data, which made adjustment impossible (Figure 1). Detailed information regarding the exact number of participants and their demographic characteristics is provided in Table 1.

**TABLE 1:** Demographic characteristics of participants.

		*C9orf72* expansion carriers		*GRN* mutation carriers		*MAPT* mutation carriers	
	Non‐carriers (Healthy control)	Presymptomatic	Symptomatic	Presymptomatic	Symptomatic	Presymptomatic	Symptomatic
N	298	129	77	137	45	50	27
Age (years)	47.3 ± 13.6	44.9 ± 11.1^a,b,c,d^	65.0 ± 7.6^a,b,e,f,g^	47.3 ± 12.3^a,f,h,i^	63.8 ± 8.4^a,b,c,j,k^	41.4 ± 11.2^a,b,g,i,k^	57.1 ± 9.6^a,b,d,e,h,j^
Mean WMH ± SD WMH (mL)	6.105 ± 15.06	7.587^c^ ± 15.31	8.673^b^ ± 8.84	6.376^a^ ± 15.72	14.446^a,b,c^ ± 18.77	11.182 ± 27.02	16.839 ± 34.21
Median WMH (mL)	15.060	15.313	8.836	15.718	18.773	27.019	34.213
Mean Log_10_ WMH (mm3)	3.469	3.535	3.751	3.487	3.921	3.542	3.766
Sex, male (%)	40.9	40.3	64.9	36.5	42.2	38.0	59.2
Education (years)	14.4 ± 3.2	14.3 ± 2.9^a,c^	12.9 ± 3.7^a,b,f^	14.8 ± 3.4^a,f,h^	11.9 ± 3.5^a,b,c,k^	14.0 ± 3.4^k^	13.3 ± 3.5^h^
NfL availability	77.8%	77.5%	67.5%	83.9%	80.0%	86.0%	81.5%
GFAP availability	61.1%	63.6%	57.1%	61.3%	66.6%	64.0%	66.6%

WMH values include raw volumes (reported as mean ± SD in mL, and median in mL) and log10‐transformed mean volumes (in cubic millimeters [mm^3^]). These values are not adjusted for age or sex. Significant differences are indicated by letters: **a** (between symptomatic and presymptomatic of that mutation group), **b** (between healthy control and this group), **c** (between presymptomatic *C9orf72* and symptomatic *GRN*), **d** (between presymptomatic *C9orf72* and symptomatic *MAPT*), **e** (between symptomatic *C9orf72* and symptomatic *MAPT*), **f** (between symptomatic *C9orf72* and presymptomatic *GRN*), **g** (between symptomatic *C9orf72* and presymptomatic *MAPT*), **h** (between presymptomatic *GRN* and symptomatic *MAPT*), **i** (between presymptomatic *GRN* and presymptomatic *MAPT*), **j** (between symptomatic *GRN* and symptomatic *MAPT*), and **k** (between symptomatic *GRN* and presymptomatic *MAPT*).

Kruskal–Wallis variance analysis was performed to compare adjusted WMH values across the three mutation cohorts and different disease stages (symptomatic and presymptomatic). Dunn's post‐hoc test, a nonparametric pairwise multiple comparison test, was conducted to determine significant differences in adjusted WMH volume between mutation groups. A similar analysis was performed for adjusted WMH volumes in each of the eight lobes. All *p*‐values reported in the manuscript are corrected for multiple comparisons with Bonferroni's method.

To rule out confounding influences on WMH, cardiovascular risk factors (stroke, hypertension, hypercholesterolemia, and diabetes) and history of traumatic brain injury were compared across mutation groups and controls (Table S1). Since no significant differences were found, these risk factor variables were not included as covariates in the model.

### 2.4 Temporal relationship analysis

After investigating the prevalence of WMH across distinct genetic groups, we aimed to determine the temporal sequence of WMH accumulation in relation to other key imaging biomarkers in groups exhibiting substantial WMH burden. In addition to lobar WMH and WMH of the whole brain, we included essential gray matter volumes and subcortical measures in FTD, i.e., frontal and temporal gray matter, cingulate, insula, cerebellum, basal ganglia (nucleus accumbens, caudate, putamen, and globus pallidus), hippocampus, amygdala, and thalamus, alongside additional biomarkers like ventricle volume, GFAP, and NfL for ranking.

#### 2.4.1 Cross‐sectional analysis using discriminative event‐based modeling

We utilized the Discriminative Event‐Based Modeling (DEBM) approach^22^, ^23^ to investigate the order of biomarker abnormalities in presymptomatic and symptomatic FTD. DEBM was well suited for our purpose because it requires only cross‐sectional data and effectively handles missing values. In DEBM, an “event” refers to the transition of a biomarker from a normal to an abnormal state, with the total number of events in disease progression corresponding to the number of biomarkers. To ensure a more normalized distribution, blood biomarkers (NfL and GFAP), ventricle volume, and WMHs were log‐transformed, effectively reducing skewness in their distributions. To control for confounding factors, the DEBM analysis incorporated sex and age by adjusting biomarker values based on these factors prior to Gaussian Mixture Modeling (GMM).

The DEBM procedure initially determines the distribution of normal and abnormal biomarker values through GMM. Using these distributions, it computes the probability for each participant that the biomarker is abnormal. This probability signifies the progression of that biomarker. Therefore, based on these probabilities, we create an approximate sequence of biomarker abnormality for each participant, which is aggregated across participants to create a robust central biomarker ordering that minimizes the sum of distances to all participant‐wise orderings.

Healthy controls, presymptomatic carriers, and symptomatic carriers were treated as distinct diagnoses to effectively model the progression. The degree of uncertainty in biomarker ordering was assessed by estimating it for 100 independently sampled datasets using bootstrap resampling with replacement. The analysis included 480 participants with verified imaging biomarkers.

For the DEBM approach to be effectively applied, two key assumptions must be considered when selecting biomarkers:

1. The biomarker must exhibit a statistically significant difference between the FTD and healthy control groups.
2. The Gaussian mixture model must be fitted appropriately, which we verified by calculating the mean squared error of the fitted distribution for each biomarker.

We rigorously assessed the accuracy of the Gaussian mixture model by calculating the mean squared error and visually inspecting the distribution. The limited sample size constrained the applicability of the DEBM approach to certain biomarkers. Biomarkers that did not meet these criteria were excluded from the DEBM analysis and subsequently evaluated using the longitudinal approach. This ensured the incorporation of only reliable biomarkers into the DEBM while allowing further investigation of excluded biomarkers through complementary methods. This integrated approach enabled us to maximize the utility of both cross‐sectional and longitudinal data, providing a comprehensive understanding of biomarker dynamics.

To validate the accuracy of the model, we assessed the disease stage that the DEBM estimated for each individual. This was done by comparing the abnormality probabilities of biomarkers for each participant with the central biomarker ordering derived from the model. Two validation approaches were employed. First, we evaluated the model's performance by calculating the area under the curve (AUC) to differentiate between symptomatic carriers and healthy controls based on estimated disease stage using 10‐fold cross‐validation. Secondly, as a construct validation metric, we examined the correlation between the estimated disease stages and key clinical scores commonly used in FTD assessment. These included the Clinical Dementia Rating‐Frontotemporal Lobar Degeneration Sum‐of‐Boxes (CDR‐FTLD SoB), Mini‐Mental State Examination (MMSE), Trail Making Test Part B (TMT‐B), Boston Naming Test, Digit Symbol substitution test, and Verbal Fluency test. Non‐parametric Spearman's rank correlation analyses were conducted to assess the relationships between the estimated disease stages and key clinical scores, given the non‐normal distribution of the overall population on these metrics. These validation metrics allowed us to assess both the discriminative power and clinical relevance of the estimated disease stages.

#### 2.4.2 Longitudinal analysis using linear regression modeling

Subsequently, we conducted a longitudinal assessment focusing on the interplay between WMHs and other neuroimaging biomarkers that were not evaluated through DEBM analysis, specifically in *GRN* mutation carriers. These biomarkers include the insula, basal ganglia (nucleus accumbens, caudate, putamen, and globus pallidus), thalamus, hippocampus, amygdala, and cingulate volumes. The process involved standardizing each biomarker to a *z*‐score by subtracting its mean and dividing by its standard deviation across the full dataset and employing linear regression models to predict the shift in each biomarker between baseline and follow‐up measurements, with WMHs serving as either the predictor or the response biomarker in each model. This approach aimed to explore directional associations between WMH and atrophy, rather than to maximize prediction accuracy. The models were designed to account for potential influences from age, sex, education, NfL, and the baseline value of the response biomarker. NfL was included to control for individual differences in global disease activity that may confound MRI‐based associations. The analysis was represented by Equation 2:

(2) $$\begin{array}{ccc} \frac{\Delta \;\mathrm{Response}\;\mathrm{biomarker}}{\Delta \;t} & = & \mathrm{Predictor}\;\mathrm{biomarke}r_{\mathrm{baseline}}\\ & & +\;\mathrm{Age}\;+\;\mathrm{Sex}+\mathrm{NfL}+\mathrm{Education}\\ & & +\;\mathrm{Response}\;\mathrm{biomarke}r_{\mathrm{baseline}} \end{array}$$

Here, $\frac{\Delta \;\mathrm{Response}\;\mathrm{biomarker}}{\Delta \;t}$ represents the predicted rate of change in the response biomarker between the baseline measurement and all subsequent follow‐up evaluations. In our analysis, WMH volumes and NfL biomarkers were log‐transformed to achieve a normal distribution.

## 3 RESULTS

### 3.1 Participant demographics and clinical information

Table 1 provides an overview of demographic and clinical data. The research included 298 family controls who did not carry the mutation. Among the 465 participants identified with mutations, *C9orf72* mutations were the most prevalent, affecting 44.3% of the group, followed by *GRN* mutations at 39.1% and *MAPT* mutations at 16.6%. Notably, 68% of these individuals were in the asymptomatic stage across the genetic variants mentioned. Among symptomatic participants, the predominant diagnosis was bvFTD, representing 66.4% of cases, with PPA at 16.8%, and amyotrophic lateral sclerosis (ALS) or FTD‐ALS at 10.7%. The remaining 6.1% of cases were diagnosed with other clinical syndromes.

Demographic analyses confirmed that symptomatic mutation carriers were older and had received fewer years of education than both presymptomatic mutation carriers and control groups (*p* < 0.001). *MAPT* mutation carriers and the control group were younger than those with the *GRN* mutation (*MAPT*: *p* = 0.01; controls: *p* = 0.001) and those carrying the *C9orf72* mutation (*MAPT*: *p *= 0.002; controls: *p *< 0.001). Additionally, symptomatic *C9orf72* mutation carriers had fewer years of education compared to the control group (*p* = 0.03). The gender ratio among symptomatic carriers also showed a higher proportion of males compared to the presymptomatic (*p* < 0.001) and control groups (*p* = 0.002). Moreover, the *C9orf72* symptomatic carrier cohort included a significantly higher proportion of males compared to *GRN* symptomatic carriers (*p* = 0.02). Other demographic characteristics remained consistent across all groups. NfL samples were obtained from 78.64% of participants, and GFAP data were available for 61.86%.

### 3.2 White matter hyperintensities across genetic groups

**FIGURE 2:**
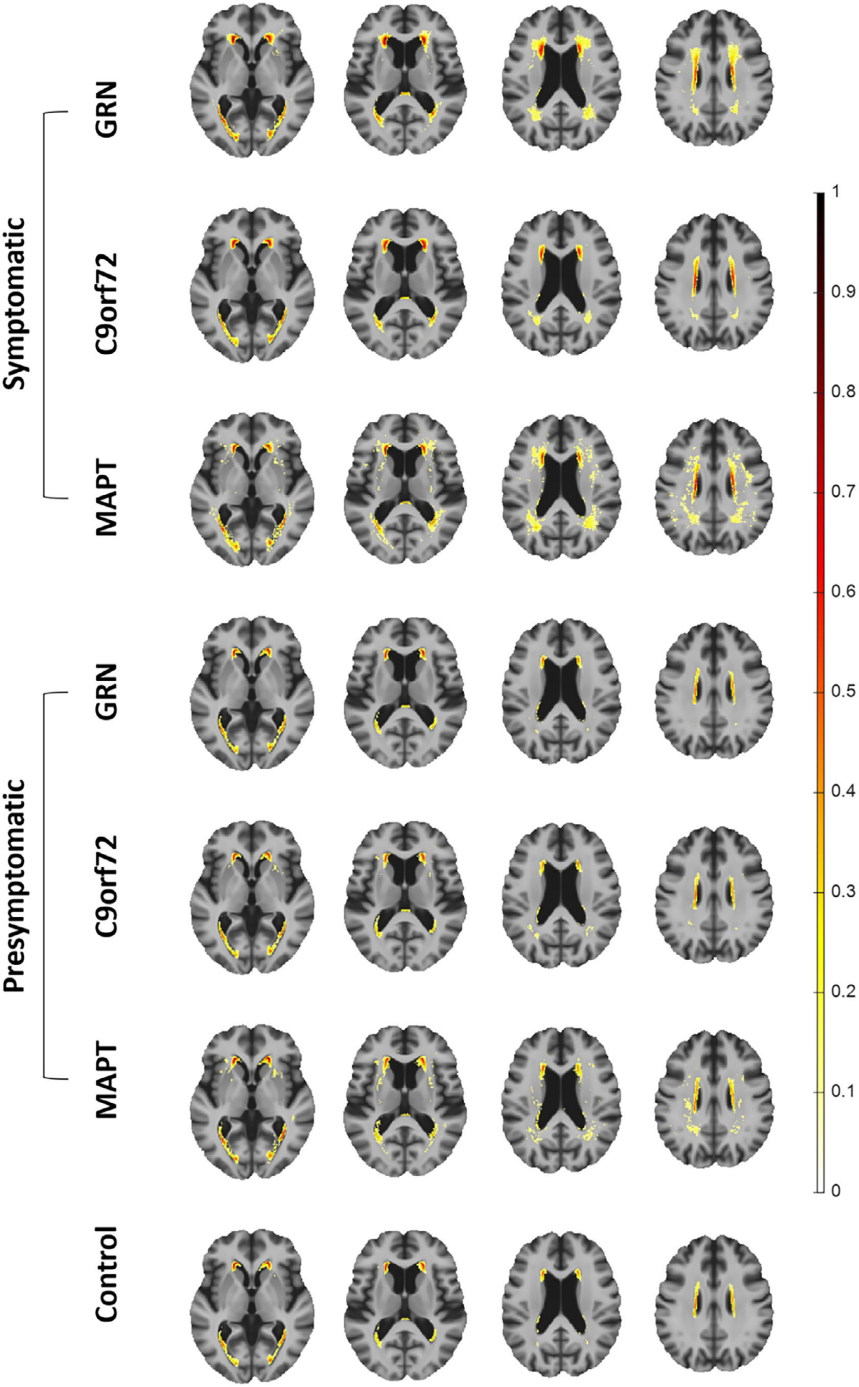
Voxel‐wise distribution of white matter hyperintensity (WMH) prevalence in mutation groups. The color bar represents the proportion of participants within each cohort exhibiting WMHs at specific voxel locations.

The WMH distribution maps in Figure 2 depict the absolute prevalence and regional distribution of WMH across mutation groups on a voxel‐wise basis. As expected, in all participants (including controls), WMHs are predominantly located in the periventricular regions, with a visually wider spatial extent in symptomatic participants.

**FIGURE 3:**
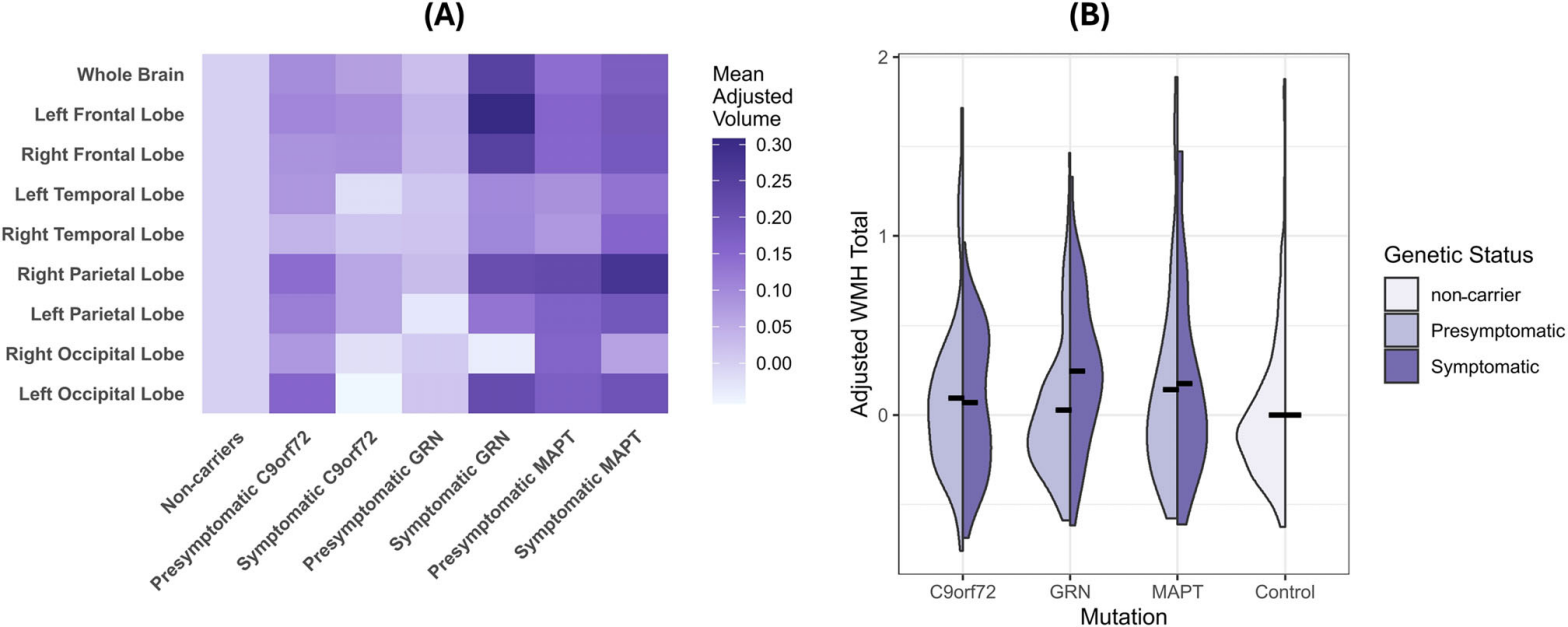
Regional and total white matter hyperintensity (WMH) volume across genetic groups. (A) Heatmap showing mean age‐ and sex‐adjusted WMH volume (log‐transformed) across brain regions and clinical/genetic subgroups. Values represent group‐level averages of residualized WMH volumes. (B) Violin plots of total age‐ and sex‐adjusted WMH volume (log‐transformed) across mutation groups and controls, stratified by clinical status. Black horizontal bars indicate group means. Among symptomatic individuals, *GRN* carriers exhibited significantly higher WMH volumes compared to controls (*P*_Bonferroni _< 0.001); no other between‐group differences reached statistical significance. Full statistical results are reported in Tables S4 and S5.

Figure 3 presents the statistical comparisons of adjusted volumes of WMH, as described in the methods section. These values are log‐transformed initially and then controlled for age and sex. For precise measures, refer to Tables S2 and S3 in the supplementary materials; these values depict the contrast of each cohort compared to the control group. Adjusted WMH volumes were significantly higher among all‐group symptomatic mutation carriers as a group compared to controls (*P*_Bonferroni_ < 0.001, *δ* = 0.215). Specifically, post hoc testing showed that the effect was driven by symptomatic carriers with *GRN* mutations who exhibited markedly elevated whole‐brain WMH volumes compared to controls (*P*_Bonferroni_ < 0.001, *δ* = 0.389), while the difference was not present for symptomatic *C9orf72* and *MAPT*. As illustrated in Figure S1, some individuals in the *MAPT* group exhibited elevated WMH volumes; however, these values did not translate into statistically or clinically meaningful group‐level effects. Among symptomatic cases, *GRN* carriers also showed higher WMH volumes compared to those with *C9orf72* mutations (*p* = 0.035); however, this difference did not remain significant after Bonferroni correction. Within the *GRN* mutation carriers, a distinctive pattern emerged, as symptomatic cases exhibited higher WMH volumes than presymptomatic *GRN* cases (*P*_Bonferroni_ = 0.001, *δ* = 0.341), underscoring the progressive nature of WMH accumulation over the disease course. Aside from the *GRN* mutation carriers, no other significant differences in whole‐brain adjusted WMH volumes were observed between the other mutation groups and controls or presymptomatic carriers.

Examination of WMH volumes per lobe revealed that the frontal lobe was the most prominent site of accumulation. Notably, symptomatic carriers demonstrated a marked increase in adjusted WMHs compared to healthy controls in both the left frontal lobe (*P*_Bonferroni_ < 0.001, *δ* = 0.26) and the right frontal lobe (*P*_Bonferroni_ < 0.001, *δ* = 0.214). Symptomatic *GRN* carriers exhibited substantially elevated WMHs in the left frontal lobe compared to controls (*P*_Bonferroni_ < 0.001, *δ* = 0.469) and symptomatic *C9orf72* (*P*_Bonferroni_ = 0.019, *δ* = 0.299). Additionally, WMH volumes in the left frontal lobe were significantly higher in symptomatic *GRN* carriers than in their presymptomatic counterparts (*P*_Bonferroni_ < 0.001, *δ* = 0.406).

A stepwise pattern of WMH increase was observed in the left frontal lobe across disease progression: presymptomatic carriers had higher WMH volumes than controls (*P*_Bonferroni_ = 0.036, *δ* = 0.118), and symptomatic carriers showed higher volumes than presymptomatic carriers (*P*_Bonferroni_ = 0.035, *δ* = 0.147). Moreover, presymptomatic *C9orf72* carriers exhibited slightly higher WMH volumes in the left frontal lobe compared to controls (*P*_Bonferroni_ = 0.027, *δ* = 0.164).

The pattern was also present in the right frontal lobe, where symptomatic *GRN* mutation carriers exhibited heightened WMHs compared to controls (*P*_Bonferroni_ < 0.001, *δ* = 0.341) and presymptomatic *GRN* carriers (*P*_Bonferroni_ = 0.009, *δ* = 0.297). There was an unexpected trend for high WMHs in the left occipital lobe of presymptomatic individuals across all genetic groups compared to controls (*P*_Bonferroni_ = 0.039, *δ *= 0.115). This effect was primarily driven by presymptomatic *C9orf72* carriers (*P*_Bonferroni_ = 0.039, *δ *= 0.15). Elevated WMH volumes were also detected in the right parietal lobe of symptomatic carriers compared to controls (*P*_Bonferroni_ = 0.017, *δ* = 0.157), particularly among those with *GRN* mutations (*P*_Bonferroni_ = 0.028, *δ* = 0.242). A complete list of these comparisons is provided in Tables S4 and S5 of the supplementary materials.

We found no significant differences in adjusted WMH volume across clinical phenotypes (bvFTD, PPA, and ALS); see Tables S6 and S7 and Figure S2 for details. As a final step, we explored the possibility of testing per *GRN* mutation subtype. The number of cases per subtype of *GRN* mutation was too small to compare prevalence across them, but we provide the adjusted level of WMH per mutation subtype in Figure S3.

### 3.3 Biomarker dynamics in *GRN* cohort

#### 3.3.1 Temporal cascade of biomarker abnormalities

**FIGURE 4:**
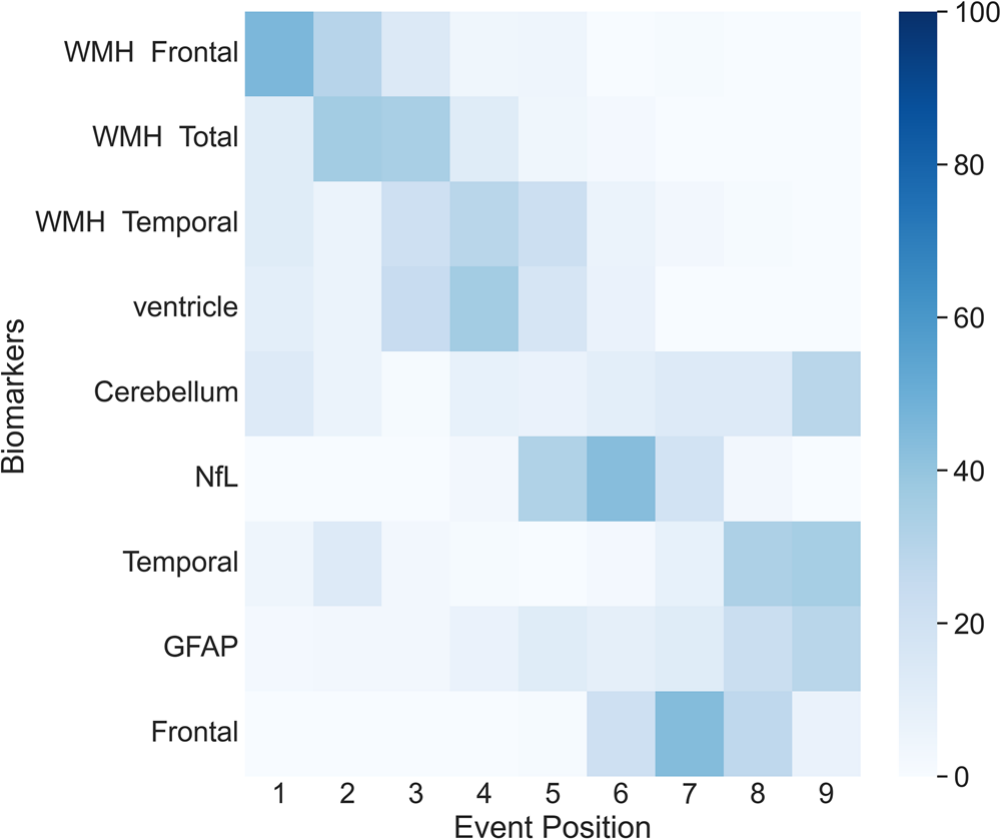
Sequence of biomarker abnormalities. The positional variance diagram for the *GRN* cohort illustrates the most probable sequence of biomarker abnormalities along with their corresponding uncertainties. The *y*‐axis (from top to bottom) orders the biomarkers by the most likely sequence as estimated by the Discriminative Event‐Based Modeling model, while the *x*‐axis indicates the position of each biomarker in the sequence, ranging from one to the total number of biomarkers. The color intensity of each square represents the frequency with which a biomarker was placed at a specific position during bootstrap resampling. The spread from bootstrap resampling reflects the standard error of the distribution, representing the uncertainty in the estimated ordering. GFAP, glial fibrillary acidic protein; NfL, neurofilament light chain; WMH, white matter hyperintensity.

Since *GRN* mutation carriers were clearly the most prominent group with a significant amount of WMHs, particularly in the frontal lobe, we focused our analysis on this cohort. We examined the temporal relationships among WMHs in the frontal lobe, WMHs in the temporal lobe, WMHs in the parietal lobe, total WMHs, and other key neuroimaging biomarkers in FTD. These included frontal and temporal gray matter, insula, cerebellum, basal ganglia, thalamus, hippocampus, amygdala, and cingulate, as well as additional biomarkers, including ventricle volume, GFAP, and NfL. All of these biomarkers exhibited significant differences between FTD and control groups (as reported in Table S8). Of these, nine biomarkers met the criteria for DEBM analysis, which required both significant group differences and adequate Gaussian mixture model fitting as assessed by the mean squared error of the Gaussian mixture model distributions (Table S9). These nine biomarkers included WMHs in the frontal lobe, WMHs in the temporal lobe, total WMHs, ventricle volume, cerebellum, frontal and temporal gray matter, and levels of GFAP and NfL. The DEBM analysis was performed on this subset of biomarkers to delineate their sequence of abnormalities and associated uncertainties in *GRN*‐associated FTD, as depicted in Figure 4. This variability was measured through 100 bootstrapping iterations (with replacement).

According to the findings depicted in Figure 4, WMH abnormalities emerged at earlier disease stages compared to other studied neuroimaging biomarkers. This initial phase of WMH changes was followed by abnormalities in ventricular size and NfL levels, which were subsequently succeeded by gray matter atrophy in the temporal and frontal lobes.

#### 3.3.2 Validation of disease stage estimation

The accuracy of estimated event ordering, validated through disease stage differentiation between symptomatic carriers and healthy controls, demonstrates robust clustering ability. This was reflected in high AUC values for distinguishing controls from *GRN* mutation carriers, with an AUC of 0.92 ± 0.05. Further validation is provided by the strong correlation between estimated disease stages and clinical scores (including CDR‐FTLD SoB, MMSE, TMT‐B, Boston Naming Test, Digit Symbol, and VF), as detailed in Table S10.

### 3.4 Longitudinal study in *GRN* mutation carriers

To further investigate the temporal relationships among biomarkers that were not included in the DEBM analysis, we conducted a longitudinal assessment focusing on *GRN* mutation carriers. This analysis explored the dynamic interplay between WMHs and other neuroimaging biomarkers, including the insula, basal ganglia (nucleus accumbens, caudate, putamen, and globus pallidus), thalamus, hippocampus, amygdala, and cingulate volumes. By quantifying *z*‐scores for each biomarker and employing linear regression models, we assessed whether changes in WMHs predicted alterations in these subcortical regions or vice versa over time. The longitudinal analysis encompassed 83 participants (70 presymptomatic and 13 symptomatic *GRN* carriers) who had follow‐up scans, enabling us to better understand how WMH changes correlate with downstream neurodegeneration. Demographic and biomarker characteristics of the longitudinal cohort are summarized in Table S11.

**FIGURE 5:**
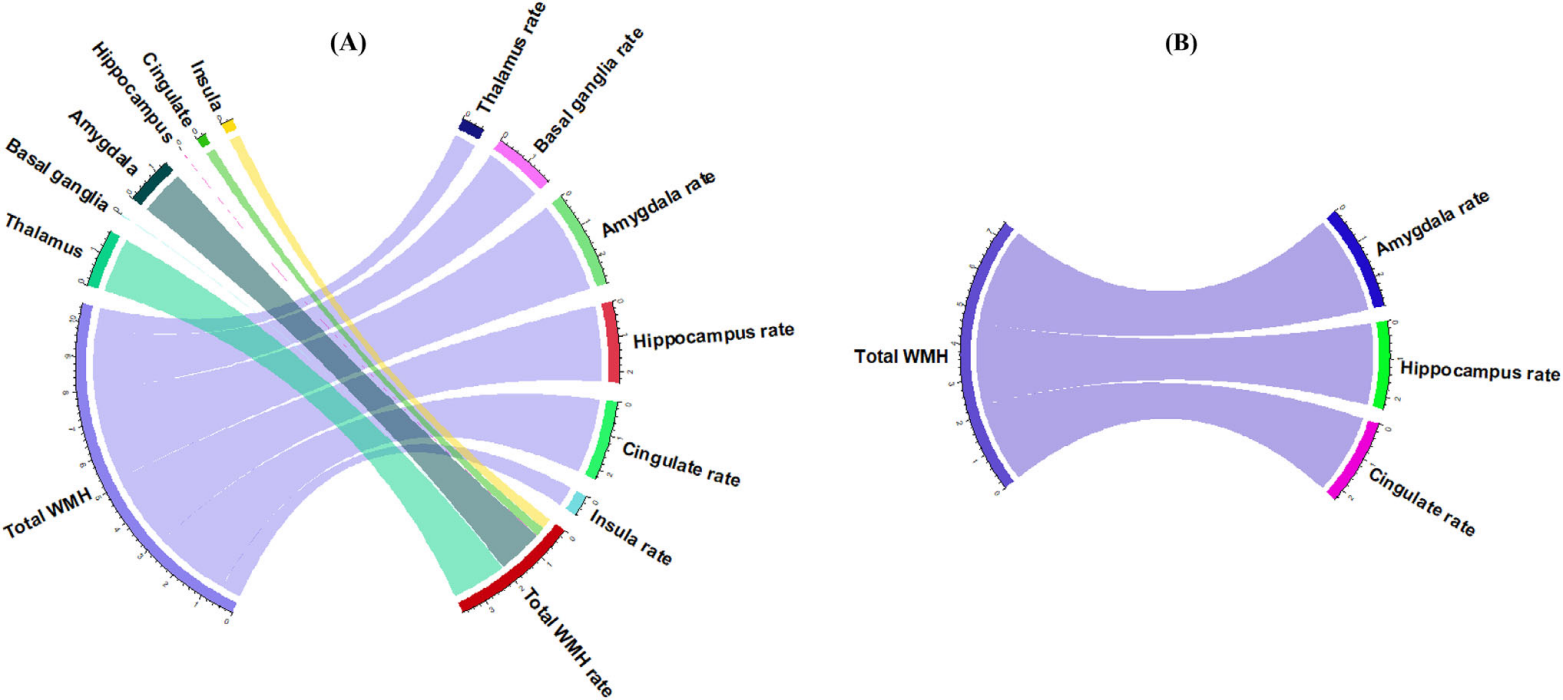
Associations between white matter hyperintensity (WMH) and subcortical biomarkers and predictability of longitudinal variations. Diagrams illustrate the associations between predictor biomarkers (left side) and the rate of change of response biomarkers (right side). The width of the connecting lines represents the *t*‐statistic, indicating the strength of the predictive association. (A) Chord diagram showing *t*‐statistics for all tests, including both significant and non‐significant associations. (B) Diagram displaying only the significant associations (FDR‐corrected *p* value < 0.05). Higher baseline WMH volumes are associated with faster volume decline in the amygdala, hippocampus, and cingulate cortex.

The estimated parameters of each model are reported in Table S12 and S13. Figure 5A, generated using the circlize package in R,^24^ displays the *t*‐statistics for all associations, regardless of significance, among the neuroimaging biomarkers identified in our analysis. Figure 5B focuses on significant associations (FDR corrected *p*‐value < 0.05), where a line connecting the baseline of a predictor biomarker on the left to the rate of change of a response biomarker on the right indicates that the predictor biomarker has predictive value for explaining variations in the response biomarker over time.

**FIGURE 6:**
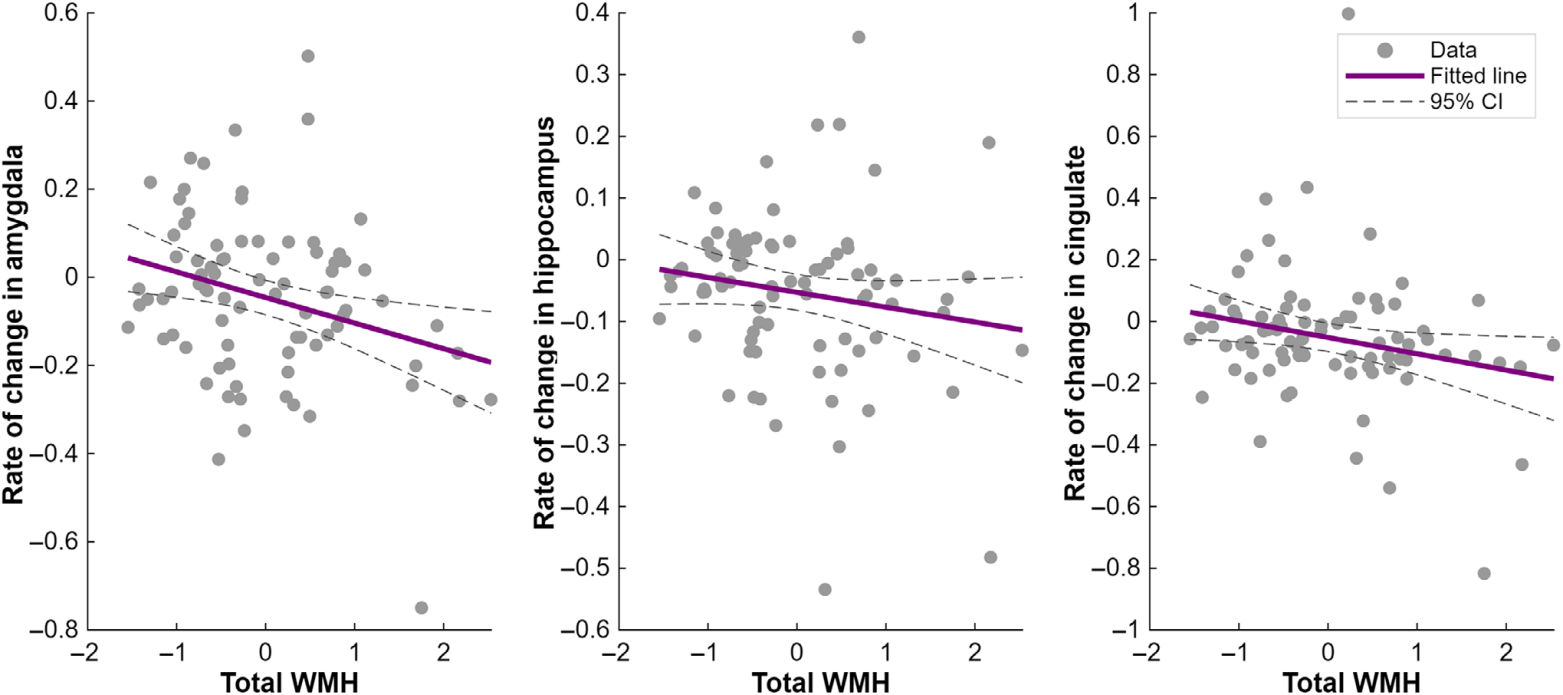
Relationship of baseline WMH volume to subsequent subcortical atrophy progression. Each scatter plot corresponds to one of the significant associations identified in Figure 5B, with baseline WMH volume (log‐transformed and *z*‐scored across the full cohort) on the *x*‐axis and the annualized rate of change in subcortical volume (total intracranial volume‐normalized and *z*‐scored prior to slope estimation) on the *y*‐axis. Linear regression lines with 95% confidence intervals illustrate the relationships.

The results shown in Figure 5B indicate that baseline WMH volumes predict subsequent brain changes. Higher baseline WMH volumes were associated with a more rapid decrease in amygdala volume (*p* = 0.006, FDR‐corrected *p* = 0.038), accelerated hippocampal atrophy (*p* = 0.023, FDR‐corrected *p* = 0.050), and faster cingulate volume reduction (*p* = 0.025, FDR‐corrected *p* = 0.050). Figure 6 presents scatter plots for these significant associations, showing the relationship between baseline WMH burden and rates of subcortical volume decline, with regression lines and 95% confidence intervals highlighting the observed trends. Interestingly, none of the studied subcortical biomarkers (thalamus, basal ganglia, amygdala, hippocampus, cingulate, and insula) were found to predict variations in WMH volumes. Our results underscore that WMHs are significant predictors of greater gray matter loss over time, emphasizing the impactful role of WMHs on brain structure changes.

## 4 DISCUSSION

Our investigation into WMHs across genetic groups in the context of FTD revealed intriguing patterns. Notably, symptomatic *GRN* mutation carriers exhibited the most pronounced WMH burden after adjusting for age and sex, with a spatial distribution concentrated in the frontal lobes and, secondarily, in parietal areas. While not present in all patients, WMHs in the *GRN* cohort followed a consistent temporal pattern, emerging early in the disease course and preceding frontal and temporal cortical atrophy. Additionally, we observed modest but significant WMH increases in the left frontal and occipital lobes of presymptomatic *C9orf72* carriers, suggesting subtle early changes in this group, but these did not translate into increased WMH burden at the symptomatic stage. Finally, although some individuals with *MAPT* mutations showed elevated WMHs, these did not result in significant group‐level effects. Collectively, our findings emphasize the distinct prominence of WMHs in *GRN*‐associated FTD.

Our results underscore the specific association of WMHs with the *GRN* mutation in genetic FTD. This finding is in line with Sudre et al.,^7^, ^8^ who also observed a similar association in a study conducted on the previous smaller GENFI data release. Patients with *GRN* mutations are deficient in progranulin, a protein that plays a crucial role in regulating the growth and survival of brain cells. There is a well‐documented link between progranulin deficiency and neuroinflammation,^25^, ^26^ and neuroinflammation is implicated in the pathogenesis of WMHs.^27^ This observation prompts a deeper exploration of how neuroinflammation contributes to the formation of WMH in the context of *GRN*‐associated FTD, providing a potential avenue for targeted therapeutic interventions.

Our DEBM analysis elucidates a sequential pattern of biomarker abnormalities in *GRN*‐associated FTD, beginning with abnormalities in WMHs. These early indicators of disease progression are subsequently followed by ventricular abnormalities and alterations in NfL levels, eventually leading to measurable temporal and frontal gray matter atrophy in later stages. This trajectory highlights the pivotal role of WMHs in understanding *GRN*‐associated FTD progression.

Our longitudinal analysis of *GRN* carriers reveals a noteworthy association between the initial volume of WMHs and subsequent reductions in gray matter volume across several critical brain regions, including the amygdala, hippocampus, and cingulate. This observation underscores the association between baseline WMHs and future neurological deterioration, characterized by pronounced atrophy within these areas. Conversely, our study found no evidence to suggest that alterations in subcortical biomarkers could serve as predictors for changes in WMH volume. Given the directional relationship observed, our study suggests that WMHs might precede the atrophy of gray matter, a conclusion that finds resonance in what was found in several studies of Alzheimer's disease.^28^, ^29^, ^30^

Furthermore, our analysis indicates that NfL abnormalities precede frontal and temporal lobe atrophy, echoing the findings of Panman et al.^23^ and Staffaroni et al.,^10^ who identified NfL as an early abnormal biomarker in the *GRN* mutation group, preceding changes in gray matter volumes, white matter microstructures, and cognitive markers. While Panman et al.^23^ identified NfL as the earliest biomarker among the key markers they investigated in FTD, their study did not include WMHs. In contrast, our findings suggest that WMHs may precede even NfL abnormalities, indicating that WMHs could represent the earliest detectable biomarker in *GRN*‐associated FTD.

We also observed that abnormalities in GFAP manifested in the late stages of the disease, while abnormalities in NfL appeared earlier. This finding is consistent with the sequence of fluid biomarkers in FTD reported by van der Ende et al.^31^

The current paradigm for modeling disease progression in genetic FTD due to *GRN* mutation may benefit from integrating WMHs into existing frameworks. Our DEBM and longitudinal analysis findings indicate early white matter disruption in FTD. A prior study by McKenna et al.^32^ highlighted alterations in white matter as relatively precise and early radiological markers, particularly effective in differentiating presymptomatic mutation carriers from healthy controls. Incorporating WMHs into disease models may offer a more comprehensive understanding of the progression from presymptomatic to symptomatic stages, shedding light on the nuanced temporal dynamics of WMH accumulation in the context of genetic FTD.

Our findings align with neuropathological evidence showing early white matter involvement in FTLD‐*GRN*. A recent study found severe frontal myelin loss in *GRN* mutation carriers, independent of axonal degeneration, suggesting a primary myelin defect.^33^ This supports our observation that WMHs appear earlier than cortical atrophy and NfL changes. Their findings also implicate microglial dysfunction and TMEM106B pathology, highlighting distinct pathogenic mechanisms and reinforcing the value of white matter biomarkers in disease staging and therapy monitoring.

In our analyses, we did not find laterality differences in *GRN* mutations at the group level (Table S14). However, it is likely that at the individual level, some subjects have predominant right‐ or left‐sided changes, which might relate to symptomatic presentation.^34^

It is essential to acknowledge certain limitations in our study. One limitation is that we used cortical volume as a measure of gray matter atrophy, which might not be as sensitive to subtle gray matter changes as cortical surface‐based measures. Additionally, the age difference between groups, where presymptomatic cases were generally younger than symptomatic ones, represents a limitation that could bias our comparisons. Given the small size of FTD datasets, balancing our groups by exclusion was not feasible. However, we accounted for age in all our analyses and modeling as a confounding factor and attempted to regress out its impact. By doing so, we aimed to minimize potential age‐related biases in our results. To further address this concern, we performed two complementary sensitivity analyses to improve age comparability between symptomatic carriers and controls. These analyses (detailed in Tables S15 and S16) used both nearest‐neighbor matching and group‐level age restriction approaches. In both cases, *GRN* carriers continued to show significantly elevated WMH volumes compared to age‐matched controls, confirming that this key result is not driven by age imbalance.

Another limitation involves scanner‐related variability inherent in multisite studies such as GENFI. Despite efforts to harmonize acquisition protocols across sites, residual differences in scanner hardware, software, or upgrades may still influence WMH quantification. We were not able to fully account for this variability due to small sample sizes at many sites; however, unmeasured scanner‐specific effects may persist and could partially impact our results.

It should be mentioned that presymptomatic cases in GENFI could include some participants with minimal or ambiguous symptoms compatible with a prodrome of FTD; however, 79.3% of cases had a confirmed CDR‐FTLD SoB of 0.

Another important limitation is the relatively small number of symptomatic cases, particularly when compared to studies applying DEBM to diseases such as Alzheimer's disease. This smaller sample size may contribute to challenges in GMM, including occasional instability in biomarker modeling. Such instability is influenced, in part, by the overlap between normal and abnormal Gaussian distributions, which becomes more pronounced when samples with abnormal biomarker values are limited. However, our study represents one of the largest cohorts available for FTD research, surpassing the size of previous reports and enhancing the reliability and generalizability of our findings.

Our DEBM results in *GRN* suggest that WMHs may precede, on average, changes in other biomarkers such as NfL or atrophy. While we did not perform detailed analyses of presymptomatic trajectories for biomarkers other than WMHs, these findings highlight the potential challenge of identifying presymptomatic *GRN* carriers at imminent risk of phenoconversion for clinical trials. This pattern contrasts with some prior models in other diseases, such as Alzheimer's disease, and underscores the heterogeneity in biomarker dynamics across neurodegenerative syndromes.

While many neuroimaging biomarkers could be explored, we focused on a curated list identified in the literature as relevant to FTD. Notably, in DEBM, adding more biomarkers can reduce certainty in the event order, emphasizing the need for deliberate selection.

Our study boasts several strengths that contribute to the robustness of our findings in addition to the larger number of participants compared to previous reports. The quality of our WMH pipeline, which remains robust across multisite data acquisition and functions effectively without the need for Fluid‐Attenuated Inversion Recovery MRI modality, further solidifies the reliability of our WMH measurements. The application of a robust event‐based modeling approach, resilient to missing values, provides a comprehensive understanding of the sequencing of biomarker abnormalities across different mutation groups.

In conclusion, our study not only contributes valuable insights into the distribution and dynamics of WMH in genetic FTD but also highlights the potential role of WMHs in refining disease progression models in *GRN* mutations. It will be important to uncover the pathological differences explaining why some *GRN* carriers develop more WMHs than others. Further research in this direction may uncover novel avenues for therapeutic interventions targeting neuroinflammatory processes associated with WMHs in genetic FTD.

## CONFLICT OF INTEREST STATEMENT

The authors report no conflicts of interest. Author disclosures are available in the supporting information.

## CONSENT STATEMENT

This study received the approval of the McGill University Health Centre review ethics board (MP‐20‐2016‐2500), and all data‐collecting sites obtained their local board approval. Written informed consent was obtained from all participants, and the research was conducted according to the ethical principles outlined in the Declaration of Helsinki.

## Supporting information



Supporting Information

Supporting Information

## Data Availability

The data used in this study are part of the GENFI dataset and can be accessed upon reasonable request through the study website (www.genfi.org), subject to review and approval by the GENFI data access committee.

## FURTHER DETAILS ARE PROVIDED IN THE SUPPLEMENTARY MATERIALS

### GENFI consortium members

Annabel Nelson, Martina Bocchetta, David Cash, David L. Thomas, Emily Todd, Hanya Benotmane, Jennifer Nicholas, Kiran Samra, Rachelle Shafei, Carolyn Timberlake, Thomas Cope, Timothy Rittman, Antonella Alberici, Enrico Premi, Roberto Gasparotti, Valentina Cantoni, Emanuele Buratti, Andrea Arighi, Chiara Fenoglio, Elio Scarpini, Giorgio Fumagalli, Vittoria Borracci, Giacomina Rossi, Giorgio Giaccone, Giuseppe Di Fede, Paola Caroppo, Pietro Tiraboschi, Sara Prioni, Veronica Redaelli, David Tang‐Wai, Ekaterina Rogaeva, Miguel Castelo‐Branco, Morris Freedman, Ron Keren, Sandra Black, Sara Mitchell, Christen Shoesmith, Robart Bartha, Rosa Rademakers, Janne M. Papma, Lucia Giannini, Rick van Minkelen, Yolande Pijnenburg, Benedetta Nacmias, Camilla Ferrari, Cristina Polito, Gemma Lombardi, Valentina Bessi, Michele Veldsman, Christin Andersson, Hakan Thonberg, Linn Öijerstedt, Vesna Jelic, Paul Thompson, Tobias Langheinrich, Albert Lladó, Anna Antonell, Jaume Olives, Mircea Balasa, Nuria Bargalló, Sergi Borrego‐Ecija, Ana Verdelho, Ana Gorostidi, Jorge Villanua, Marta Cañada, Mikel Tainta, Miren Zulaica, Myriam Barandiaran, Patricia Alves, Benjamin Bender, Lisa Graf, Annick Vogels, Mathieu Vandenbulcke, Philip Van Damme, Rose Bruffaerts, Koen Poesen, Pedro Rosa‐Neto, Serge Gauthier, Anne Bertrand, Aurélie Funkiewiez, Daisy Rinaldi, Dario Saracino, Olivier Colliot, Sabrina Sayah, Catharina Prix, Elisabeth Wlasich, Olivia Wagemann, Sandra Loosli, Sonja Schönecker, Tobias Hoegen, Jolina Lombardi, Sarah Anderl‐Straub, Adeline Rollin, Gregory Kuchcinski, Maxime Bertoux, Thibaud Lebouvier, Vincent Deramecour, Beatriz Santiago, Diana Duro, Maria João Leitão, Maria Rosario Almeida, Miguel Tábuas‐Pereira, Sónia Afonso
