# Supplementary material for: White matter hyperintensities precede other biomarkers in GRN frontotemporal dementia
Source: Alzheimers Dement. 2025 Oct 7;21(10):e70695. doi: 10.1002/alz.70695 (PMC12504049; doi:10.1002/alz.70695)
Supplement: Supplementary file 1 — Supporting Information [file ALZ-21-e70695-s001.docx]

# Supplementary materials

**Table S1. Summary of cardiovascular risk factors in the studied population.** Fisher's exact tests were performed to compare the distribution of vascular factors (absent vs. present) across genetic groups, with FDR correction for multiple comparisons. Smoking data were available for fewer participants as this metric was not collected in GENFI1 (sample size indicated in the smoking section). P-value1: comparison between control and presymptomatic groups; P-value 2: comparison among symptomatic cohorts.

|  | Non-Carriers | Presymptomatic | | |  | Symptomatic | | |  |
| --- | --- | --- | --- | --- | --- | --- | --- | --- | --- |
|  |  | C9orf72 | GRN | MAPT | Adjusted P-value1  (P-value1) | C9orf72 | GRN | MAPT | Adjusted P-value2  (P-value2) |
| **N** | 297 | 128 | 136 | 50 | - | 76 | 45 | 27 | - |
| **Hypertension**  Absent  Recent/Active  Remote/Inactive | 256 (86%)  36 (12%)  5 (2%) | 107 (83.5%)  19 (15%)  2 (1.5%) | 117 (86%)  16 (12%)  3 (2%) | 44 (88%)  5 (10%)  1 (2%) | 0.88  (0.88) | 56 (74%)  19 (25%)  1 (1%) | 29 (65%)  14 (31%)  2 (4%) | 20 (74%)  7 (26%)  0 | 0.58  (0.55) |
| **Hypercholesterolemia**  Absent  Recent/Active  Remote/Inactive | 260 (87%)  32 (11%)  5 (2%) | 114 (89%)  13 (10%)  1 (1%) | 124 (91%)  11 (8%)  1 (1%) | 47 (94%)  3(6%)  0 (0%) | 0.88  (0.51) | 51 (67%)  22 (29%)  3 (4%) | 27 (60%)  16 (35.5%)  2 (4.5%) | 20 (74%)  7(26%)  0 (0%) | 0.58  (0.48) |
| **Diabetes**  Absent  Recent/Active  Remote/Inactive | 289 (97%)  5 (2%)  3 (1%) | 127 (99%)  1 (1%)  0 | 134 (98%)  2 (2%)  0 | 49 (98%)  1 (2%)  0 | 0.88  (0.63) | 72 (95%)  4 (5%)  0 | 42 (93%)  3 (7%)  0 | 24 (89%)  3 (11%)  0 | 0.58  (0.50) |
| **Stroke**  Absent  Recent/Active  Remote/Inactive | 296 (99.7%)  0  1 (0.3%) | 128 (100%)  0  0 | 132 (97%)  0  4 (3%) | 49 (98%)  0  1 (2%) | 0.25  (0.04) | 72 (95%)  1 (1%)  3 (4%) | 44 (98%)  0  1 (2%) | 27 (100%)  0  0 | 0.58  (0.58) |
| **Traumatic Brain Injury**  Absent  Recent/Active  Remote/Inactive | 264 (89%)  1 (0.3%)  32 (10.7%) | 118 (92%)  1 (1%)  9 (7%) | 123 (90%)  0  13 (10%) | 46 (92%)  1 (2%)  3 (6%) | 0.88  (0.77) | 70 (92%)  2 (3%)  4 (5%) | 39 (87%)  0  6 (13%) | 26 (96%)  0  1 (4%) | 0.58  (0.33) |
| **Smoking**  Sample size  Absent  Recent/Active  Remote/Inactive | 261  177 (68%)  36 (14%)  48 (18%) | 119  90 (76%)  10 (8%)  19 (16%) | 133  92 (69%)  19 (14%)  22 (16%) | 48  34 (71%)  8 (17%)  6 (12%) | 0.88  (0.48) | 61  40 (66%)  5 (8%)  16 (26%) | 39  24 (61%)  5 (13%)  10 (26%) | 19  15 (79%)  1 (5%)  3 (16%) | 0.58  (0.45) |

**Table S2. Average regional WMH volume across distinct mutation cohorts** (raw values in mm3)

|  | **Non-carriers (Healthy control)** | ***C9orf72* expansion carriers** | | ***GRN* mutation carriers** | | ***MAPT* mutation carriers** | |
| --- | --- | --- | --- | --- | --- | --- | --- |
|  |  | **Presymptomatic** | **Symptomatic** | **Presymptomatic** | **Symptomatic** | **Presymptomatic** | **Symptomatic** |
| Whole Brain | 6105.097 | 7587.53 | 8672.57 | 6376.52 | 14445.80 | 11181.56 | 16838.93 |
| Left Frontal Lobe | 1556.017 | 1954.209 | 2428.844 | 1562.482 | 4280.289 | 2856.280 | 4219.148 |
| Right Frontal Lobe | 1615.326 | 1928.256 | 2502.688 | 1693.847 | 4369.911 | 3090.220 | 4606.815 |
| Left Temporal Lobe | 423.0705 | 495.488 | 503.558 | 496.686 | 730.667 | 675.960 | 960.259 |
| Right Temporal Lobe | 457.611 | 505.318 | 569.013 | 505.431 | 794.089 | 739.140 | 1166.222 |
| Left Parietal Lobe | 776.164 | 1083.628 | 1038.623 | 790.226 | 1579.578 | 1549.020 | 2530.407 |
| Right Parietal Lobe | 720.255 | 989.853 | 903.156 | 742.693 | 1609.022 | 1359.740 | 2224.037 |
| Left Occipital Lobe | 222.762 | 250.512 | 369.779 | 216.474 | 513.267 | 340.480 | 571.926 |
| Right Occipital Lobe | 229.899 | 260.659 | 315.935 | 238.401 | 430.689 | 364.200 | 387.778 |

**Table S3. Average regional WMH volume across distinct mutation cohorts, with adjustments for age and sex.** The values have been subjected to a log transformation.

|  | **Non-carriers (Healthy control)** | ***C9orf72* expansion carriers** | | ***GRN* mutation carriers** | | ***MAPT* mutation carriers** | |
| --- | --- | --- | --- | --- | --- | --- | --- |
|  |  | **Presymptomatic** | **Symptomatic** | **Presymptomatic** | **Symptomatic** | **Presymptomatic** | **Symptomatic** |
| Whole Brain | 0.0000 | 0.0951 | 0.0682 | 0.0260 | 0.2446 | 0.1420 | 0.1747 |
| Left Frontal Lobe | 0.0000 | 0.1063 | 0.0962 | 0.0412 | 0.3077 | 0.1568 | 0.1895 |
| Right Frontal Lobe | 0.0000 | 0.0862 | 0.0924 | 0.0369 | 0.2447 | 0.1559 | 0.1857 |
| Left Temporal Lobe | 0.0000 | 0.0825 | -0.0198 | 0.0132 | 0.1014 | 0.0894 | 0.1337 |
| Right Temporal Lobe | 0.0000 | 0.0417 | 0.0138 | 0.0181 | 0.1063 | 0.0786 | 0.1577 |
| Left Parietal Lobe | 0.0000 | 0.1195 | 0.0608 | 0.0331 | 0.1314 | 0.1679 | 0.1910 |
| Right Parietal Lobe | 0.0000 | 0.1446 | 0.0594 | 0.0278 | 0.2128 | 0.2227 | 0.2786 |
| Left Occipital Lobe | 0.0000 | 0.1568 | -0.0558 | 0.0159 | 0.2182 | 0.1744 | 0.1999 |
| Right Occipital Lobe | 0.0000 | 0.0791 | -0.0219 | 0.0079 | -0.0407 | 0.1627 | 0.0629 |

**Table S4. Comparison of adjusted WMHs across brain lobes among different mutation groups.** Only comparisons with unadjusted p-values of ≤ 0.05 are listed. WMHs are adjusted for age and sex.

| **Region** | **Mutation types** | **Z** | **Unadjusted**  **p-value** | **Bonferroni Adjusted**  **p-value** | **FDR Adjusted**  **p-value** | **Effect Size**  **(Cliffs Delta)** | **95% CI (Cliffs Delta)** |
| --- | --- | --- | --- | --- | --- | --- | --- |
| Left Frontal lobe | Symptomatic *GRN* > control | 5.0594 | < 0.0001 | **< 0.0001** | < 0.0001 | 0.4694 | [0.3123, 0.6222] |
| Whole Brain | Symptomatic *GRN* > control | 4.1788 | < 0.0001 | **0.0001** | 0.0001 | 0.3888 | [0.2112, 0.5594] |
| Right Frontal lobe | Symptomatic *GRN* > control | 3.7047 | 0.0002 | **0.0006** | 0.0006 | 0.3412 | [0.1497, 0.5174] |
| Left Frontal lobe | Symptomatic *GRN* > Symptomatic *C9orf72* | 2.7297 | 0.0063 | **0.019** | 0.019 | 0.2993 | [0.1053, 0.4875] |
| Left Frontal lobe | Presymptomatic *C9orf72* > control | 2.6103 | 0.009 | **0.0271** | 0.0271 | 0.1636 | [0.0433, 0.2859] |
| Right Parietal | Symptomatic *GRN* > control | 2.6031 | 0.0092 | **0.0277** | 0.0277 | 0.2419 | [0.0543, 0.4269] |
| Left Occipital | Presymptomatic *C9orf72* > control | 2.4806 | 0.0131 | **0.0394** | 0.0394 | 0.1505 | [0.0351, 0.2722] |
| Left Temporal | Presymptomatic *C9orf72* > control | 2.2696 | 0.0232 | 0.0697 | 0.0697 | 0.1412 | [0.0295, 0.2668] |
| Whole Brain | Presymptomatic C9orf72 > control | 2.2628 | 0.0236 | 0.0709 | 0.0709 | 0.1428 | [0.0279, 0.2663] |
| Right Parietal | Presymptomatic *C9orf72* > control | 2.2593 | 0.0239 | 0.0716 | 0.0716 | 0.1417 | [0.0173, 0.2666] |
| Left Frontal lobe | Symptomatic *C9orf72* > control | 2.2239 | 0.0262 | 0.0785 | 0.0392 | 0.1566 | [0.0022, 0.3133] |
| Left Occipital | Symptomatic *GRN* > control | 2.2203 | 0.0264 | 0.0792 | 0.0792 | 0.2076 | [0.0375, 0.3841] |
| Right Frontal | Symptomatic *C9orf72*> control | 2.1883 | 0.0286 | 0.0859 | 0.0826 | 0.1556 | [-0.0018, 0.3152] |
| Whole Brain | Symptomatic *GRN* > Symptomatic *C9orf72* | 2.1043 | 0.0354 | 0.1061 | 0.1061 | 0.2277 | [0.0274, 0.4303] |
| Left Temporal | Symptomatic *GRN* > control | 1.9748 | 0.0483 | 0.1449 | 0.1449 | 0.1831 | [-0.0207, 0.3736] |
| Right Temporal | Symptomatic *GRN* > control | 1.9679 | 0.0491 | 0.1472 | 0.1472 | 0.1805 | [-0.0179, 0.3672] |

**Table S5. Comparison of adjusted WMHs across brain lobes among different mutation groups in different disease stages.** Only comparisons with unadjusted p-values of ≤ 0.05 are listed.

| **Region** | **Genetic Group** | **Genetic status** | **Z** | **Unadjusted p-value** | **Bonferroni Adjusted**  **p-value** | **FDR Adjusted**  **p-value** | **Effect Size**  **(Cliffs Delta)** | **95% CI**  **(Cliffs Delta)** |
| --- | --- | --- | --- | --- | --- | --- | --- | --- |
| Left Frontal | all | Symptomatic > control | 4.5176 | < 0.0001 | **< 0.0001** | < 0.0001 | 0.2599 | [0.1514, 0.3690] |
| Left Frontal | *GRN* | Symptomatic > Presymptomatic | 4.1226 | < 0.0001 | **0.0001** | 0.0001 | 0.4057 | [0.2337, 0.5734] |
| Right Frontal | all | Symptomatic > control | 3.7397 | 0.0002 | **0.0006** | 0.0006 | 0.2143 | [0.1003, 0.3313] |
| Whole brain | all | Symptomatic > control | 3.7382 | 0.0002 | **0.0006** | 0.0006 | 0.2148 | [0.0970, 0.3312] |
| Whole brain | *GRN* | Symptomatic > Presymptomatic | 3.488 | 0.0005 | **0.0015** | 0.0007 | 0.3408 | [0.1445, 0.5218] |
| Right Frontal | *GRN* | Symptomatic > Presymptomatic | 2.9614 | 0.0031 | **0.0092** | 0.0046 | 0.2967 | [0.1004, 0.4706] |
| Right Parietal | all | Symptomatic > control | 2.7684 | 0.0056 | **0.0169** | 0.0169 | 0.1572 | [0.0437, 0.2781] |
| Left Frontal | all | Symptomatic > presymptomatic | 2.5216 | 0.0117 | **0.035** | 0.0121 | 0.1467 | [0.0359, 0.2558] |
| Left Frontal | all | Presymptomatic > control | 2.51 | 0.0121 | **0.0362** | 0.0121 | 0.1180 | [0.0301, 0.2050] |
| Left Occipital | all | Presymptomatic > control | 2.4867 | 0.0129 | **0.0387** | 0.0387 | 0.1151 | [0.0283, 0.2086] |
| Right Parietal | all | Presymptomatic > control | 2.2096 | 0.0271 | 0.0814 | 0.0407 | 0.1046 | [0.0148, 0.1859] |
| Right Frontal | all | Symptomatic > Presymptomatic | 2.1465 | 0.0318 | 0.0955 | 0.0449 | 0.1256 | [0.0111, 0.2442] |
| Whole brain | all | Symptomatic > Presymptomatic | 2.1324 | 0.033 | 0.0989 | 0.0433 | 0.1242 | [0.0069, 0.2372] |
| Right Temporal | all | Symptomatic > control | 2.1039 | 0.0354 | 0.1062 | 0.1062 | 0.1200 | [0.0082, 0.2344] |
| Left Occipital | all | Symptomatic > control | 2.0305 | 0.0423 | 0.1269 | 0.0635 | 0.1197 | [0.0008, 0.2340] |
| Whole brain | all | Presymptomatic > control | 2.0206 | 0.0433 | 0.1300 | 0.0433 | 0.0951 | [0.0092, 0.1817] |
| Right Frontal | all | Presymptomatic > control | 2.0051 | 0.0449 | 0.1348 | 0.0449 | 0.0947 | [0.0061, 0.1851] |
| Right Parietal | *GRN* | Symptomatic > Presymptomatic | 1.9412 | 0.0522 | 0.1567 | 0.0784 | 0.1899 | [-0.0190, 0.3914] |

**
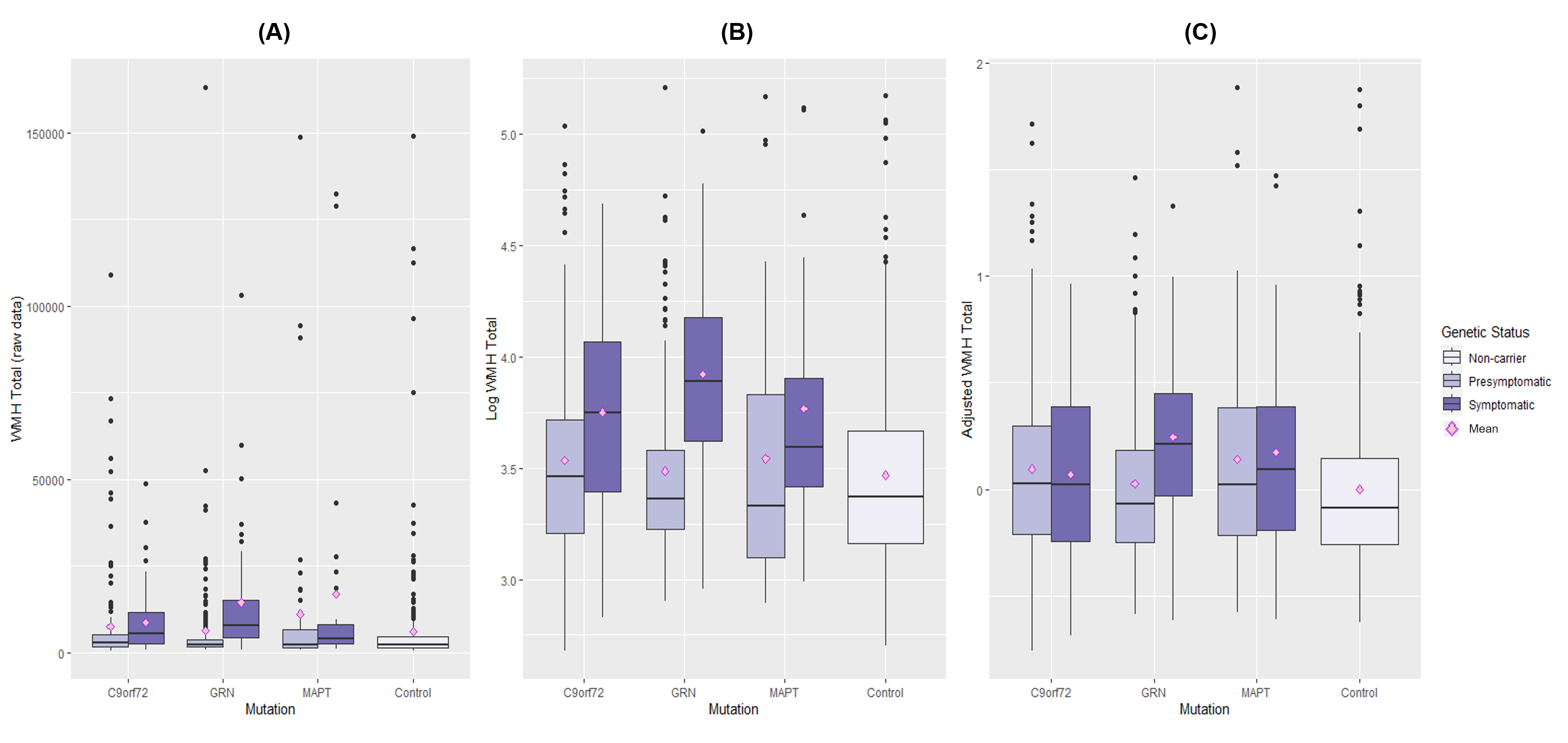
**

**Figure S1. Boxplots of WMH volumes across cohorts:** (A) raw data, (B) log-transformed values, and (C) log-transformed values adjusted for age and sex.

**Table S6:** Breakdown of clinical diagnoses for symptomatic participants within each genetic group. bvFTD: behavioral variant frontotemporal dementia; PPA: primary progressive aphasia; ALS: amyotrophic lateral sclerosis. The ALS group includes individuals with ALS and FTD-ALS diagnoses.

| **Characteristic** | **Symptomatic *C9orf72* expansion carriers** | **Symptomatic *GRN* mutation carriers** | **Symptomatic*MAPT* mutation carriers** |
| --- | --- | --- | --- |
| **N** | 77 | 45 | 27 |
| bvFTD | 53 | 22 | 24 |
| PPA | 4 | 20 | 1 |
| FTD-ALS or ALS | 16 | - | - |
| Other | 4 | 3 | 2 |

**Table S7:** Results of pairwise Dunn’s tests comparing age- and sex-adjusted total WMH volume across clinical phenotypes. No comparisons reached statistical significance after correction for multiple testing. n1-n2 shows the sample size in two compared cohorts.

| **Mutation Group** | **Compared Phenotypes** | **n1-n2** | **Z** | **Unadjusted**  **p-value** | **Bonferroni Adjusted**  **p-value** | **FDR Adjusted**  **p-value** | **Effect Size**  **(Cliffs Delta)** | **95% CI (Cliffs Delta)** |
| --- | --- | --- | --- | --- | --- | --- | --- | --- |
| All | bvFTD-PPA | 99-25 | 0.348 | 0.728 | 1 | 0.728 | 0.048 | [-0.217, 0.316] |
| All | ALS- bvFTD | 16-99 | -2.351 | 0.019 | 0.056 | 0.056 | -0.362 | [-0.664,--0.050] |
| All | ALS-PPA | 16-25 | -1.735 | 0.083 | 0.248 | 0.124 | -0.340 | [-0.675, 0.030] |
| GRN | bvFTD - PPA | 22-20 | 1.284 | 0.199 | 0.199 | 0.199 | 0.232 | [-0.127, 0.573] |
| C9orf72 | ALS- bvFTD | 16-53 | -1.725 | 0.085 | 0.254 | 0.254 | -0.281 | [-0.620, 0.097] |


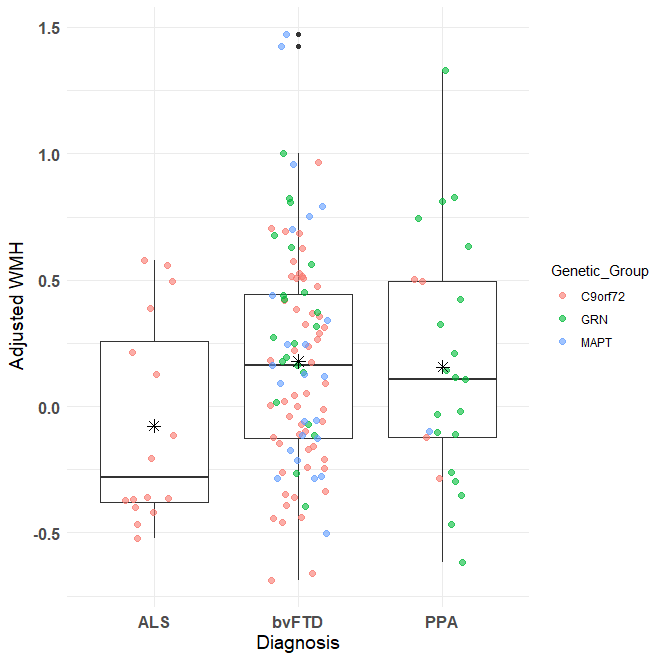


**Figure S2:** Boxplot of total WMH volume (adjusted for age and sex) in symptomatic participants with bvFTD, PPA, and ALS. bvFTD: behavioral variant frontotemporal dementia; PPA: primary progressive aphasia; ALS: amyotrophic lateral sclerosis. The ALS group includes individuals with ALS and FTD-ALS diagnoses. WMHs are adjusted for age and sex.

**
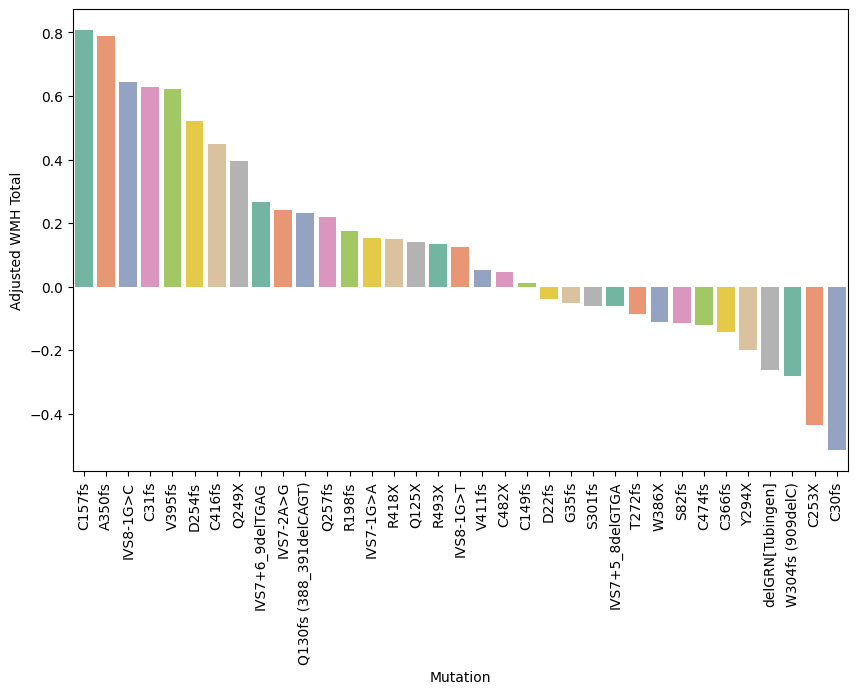
**

**Figure S3. Adjusted WMH Volumes by GRN Mutation Subtype** Each bar represents the average of adjusted WMH volumes (controlled for age, sex, and scanner site as explained in Equation 1) in different GRN mutation subtypes among all GRN carriers (both presymptomatic and symptomatic). Due to the small number of cases in certain groups and confidentiality considerations, we did not report the variability within each group or the exact number of cases per mutation subtype.

**Table S8.** **Comparison of raw biomarker values between FTD and healthy controls,** without adjustment for age and sex, to identify statistically significant differences as part of the inclusion criteria for DEBM analysis.

| **Biomarker** | **tStat** | **pVal** |
| --- | --- | --- |
| NfL | -15.025 | < 0.0001 |
| GFAP | -7.664 | < 0.0001 |
| Total WMH | -6.338 | < 0.0001 |
| WMH Frontal | -6.987 | < 0.0001 |
| WMH Temporal | -4.280 | < 0.0001 |
| WMH Parietal | -4.918 | < 0.0001 |
| Ventricle | -14.723 | < 0.0001 |
| Frontal GM | -11.606 | < 0.0001 |
| Temporal GM | -8.853 | < 0.0001 |
| Cerebellum | -5.581 | < 0.0001 |
| Insula | -15.169 | < 0.0001 |
| Basal Ganglia | -8.337 | < 0.0001 |
| Thalamus | -6.695 | < 0.0001 |
| Amygdala | -4.858 | < 0.0001 |
| Hippocampus | -6.214 | < 0.0001 |
| Cingulate | -11.128 | < 0.0001 |

**Table S9. Mean Square Error (MSE) of Gaussian Mixture Modeling in the DEBM Framework.** This table shows the MSE values for distributions fitted using Gaussian mixture modeling as part of the DEBM model. The MSE is calculated by comparing the fitted Gaussian mixture model with the histogram of the data. Biomarkers with an MSE exceeding 13 are considered poorly fitted, excluded from DEBM analysis, and included in the longitudinal analysis instead.

| **Biomarker** | **Mean Square Error** |
| --- | --- |
| NfL | 9.8408 |
| GFAP | 4.5165 |
| Total WMH | 11.5816 |
| WMH Frontal | 10.2852 |
| WMH Temporal | 6.8822 |
| WMH Parietal | 13.993 |
| Ventricle | 12.9720 |
| Frontal GM | 9.5862 |
| Temporal GM | 6.7830 |
| Cerebellum | 7.2356 |
| Insula | 67.4482 |
| Basal Ganglia | 91.9080 |
| Thalamus | 85.7825 |
| Amygdala | 174.4104 |
| Hippocampus | 77.6946 |
| Cingulate | 66.2930 |

**Table S10. Correlation between DEBM staging and clinical scores (among mutation carriers),** including Clinical Dementia Rating-Frontotemporal Lobar Degeneration Sum-of-Boxes (CDR-FTLD SoB), Mini-Mental State Examination (MMSE), Trail Making Test Part B (TMTB) time, Digit Symbol substitution test, Boston naming, and Verbal Fluency (VF) combined score.

| **Clinical score** | **Spearman's Rank Correlation** | **t** | **df** | **p-value** |
| --- | --- | --- | --- | --- |
| CDR-FTLD SoB | 0.57 | 8.97 | 170 | < 0.001 |
| MMSE | -0.48 | -6.99 | 164 | < 0.001 |
| TMTB time | 0.36 | 4.81 | 160 | < 0.001 |
| Digit symbol | -0.43 | -6.22 | 168 | < 0.001 |
| Boston naming | -0.28 | -3.88 | 171 | < 0.001 |
| VF combined | -0.40 | -5.57 | 165 | < 0.001 |

**Table S11. Demographic and biomarker characteristics of GRN mutation carriers included in the longitudinal analysis.** NfL: neurofilament light chain; WMH: white matter hyperintensities.

|  |  | ***GRN* mutation carriers** | | |
| --- | --- | --- | --- | --- |
|  |  | **Presymptomatic (n=70)** | **Symptomatic (n=13)** | **Total (n=83)** |
|  | Number of visits, range | [2,6] | [2,6] | [2,6] |
|  | Number of visits, mean ± SD | 3.29 ± 1.14 | 2.62 ± 1.19 | 3.18 ± 1.17 |
|  | Interval between visits (years), mean ± SD | 1.20 ± 0.43 | 1.06 ± 0.14 | 1.18 ± 0.41 |
|  | Sex, male (%) | 41.43% | 38.46% | 40.96% |
| **At First Visit** | Age (years),  mean ± SD | 48.00 ± 11.38 | 63.34 ± 8.07 | 50.40 ± 12.25 |
|  | Education (years), mean | 15.24 | 12.15 | 14.76 |
|  | Log NfL (pg/mL), mean ± SD | 2.13 ± 0.53 | 3.63 ± 0.77 | 2.37 ± 0.79 |
|  | WMH volume (mm3), mean ± SD | 4946.19  ± 6094.57 | 9155.38  ± 9910.76 | 5605.46  ± 6928.07 |
|  | Log WMH, mean | 8.09 | 8.66 | 8.18 |

**Table S12. Parameter estimates for the longitudinal analysis model,** where the change in subcortical regions over time (ΔResponse biomarker/Δt) is modeled as a function of baseline WMH (predictor biomarker), age, sex, neurofilament light chain (NfL), education, and baseline subcortical biomarker.

| **Predictor biomarker** | **Response biomarker** | **Predictor biomarker baseline** | | | | **Response biomarker baseline** | | **NfL** | | **Age** | | **Sex** | | **Education** | |
| --- | --- | --- | --- | --- | --- | --- | --- | --- | --- | --- | --- | --- | --- | --- | --- |
|  |  | **tStat** | **pVal** | | **FDR pVal** | **tStat** | **pVal** | **tStat** | **pVal** | **tStat** | **pVal** | **tStat** | **pVal** | **tStat** | **pVal** |
| Total WMH | Thalamus | -0.68 | 0.497 | 0.540193 | | -2.76 | 0.007 | -2.59 | 0.011 | -0.39 | 0.699 | -1.58 | 0.119 | -0.44 | 0.661 |
| Total WMH | Basal Ganglia | -1.81 | 0.074 | 0.111 | | -1.19 | 0.239 | -4.49 | < 0.001 | 1.54 | 0.127 | -0.11 | 0.915 | -1.19 | 0.236 |
| Total WMH | Amygdala | -2.80 | 0.006 | **0.038** | | -3.81 | < 0.001 | -2.11 | 0.038 | -1.07 | 0.288 | 0.44 | 0.660 | -0.42 | 0.675 |
| Total WMH | Hippocampus | -2.32 | 0.023 | **0.050** | | -3.80 | < 0.001 | -2.60 | 0.011 | -0.88 | 0.382 | 0.87 | 0.387 | -1.27 | 0.207 |
| Total WMH | Cingulate | -2.28 | 0.025 | **0.050** | | -1.71 | 0.090 | -3.29 | 0.001 | 1.12 | 0.264 | -0.51 | 0.607 | -0.54 | 0.588 |
| Total WMH | Insula | 0.61 | 0.540 | 0.540 | | 0.003 | 0.997 | -5.60 | < 0.001 | 2.30 | 0.024 | 1.61 | 0.111 | -1.33 | 0.186 |

**Table S13. Parameter estimates for the longitudinal analysis model,** where the change in WMH over time (ΔResponse biomarker/Δt) is modeled as a function of baseline predictor biomarker (subcortical volumes), age, sex, neurofilament light chain (NfL), education, and baseline WMH.

| **Predictor biomarker** | **Response biomarker** | **Predictor biomarker baseline** | | | **Response biomarker baseline** | | **NfL** | | **Age** | | **Sex** | | **Education** | |
| --- | --- | --- | --- | --- | --- | --- | --- | --- | --- | --- | --- | --- | --- | --- |
|  |  | **tStat** | **pVal** | **FDR pVal** | **tStat** | **pVal** | **tStat** | **pVal** | **tStat** | **pVal** | **tStat** | **pVal** | **tStat** | **pVal** |
| Thalamus | Total WMH | 1.67 | 0.098 | 0.497 | -1.47 | 0.144 | 1.93 | 0.057 | 0.88 | 0.380 | 0.95 | 0.345 | 0.92 | 0.359 |
| Basal Ganglia | Total WMH | -0.03 | 0.974 | 0.991 | -1.99 | 0.049 | 1.57 | 0.120 | 0.42 | 0.679 | 0.51 | 0.613 | 1.16 | 0.251 |
| Amygdala | Total WMH | 1.40 | 0.166 | 0.497 | -1.94 | 0.056 | 1.76 | 0.083 | 0.86 | 0.394 | 0.50 | 0.615 | 0.88 | 0.378 |
| Hippocampus | Total WMH | 0.01 | 0.991 | 0.991 | -1.96 | 0.054 | 1.68 | 0.097 | 0.41 | 0.680 | 0.52 | 0.606 | 1.14 | 0.258 |
| Cingulate | Total WMH | 0.28 | 0.781 | 0.991 | -1.91 | 0.060 | 1.72 | 0.088 | 0.46 | 0.648 | 0.56 | 0.577 | 1.15 | 0.255 |
| Insula | Total WMH | -0.34 | 0.732 | 0.991 | -2.06 | 0.043 | 1.44 | 0.154 | 0.34 | 0.735 | 0.43 | 0.668 | 1.18 | 0.243 |

**Table S14:** Comparison of Left and Right Hemisphere WMH Volumes in GRN Mutation Carriers

| Stage | N | Raw WMH (mm3) | | Left vs Right comparison | | |
| --- | --- | --- | --- | --- | --- | --- |
|  |  | Left  (mean± SD) | Right  (mean± SD) | Wilcoxon W | p-value | Effect size  (Rank-biserial correlation) |
| Presymptomatic | 137 | 3065.9 ± 7496.8 | 3180.4± 7495.5 | 9330.5 | 0.935 | 0.006 |
| Symptomatic | 45 | 7103.8± 9243.0 | 7203.7± 9717.1 | 1068 | 0.657 | 0.055 |

## Age-Matched Control Comparisons:

To assess the potential impact of age differences between symptomatic mutation carriers and the control group, we performed two complementary sensitivity analyses using age-matched control samples.

In the first approach, we used nearest-neighbor matching (via the *MatchIt* package in R) to pair each symptomatic carrier with a control participant of similar age at visit in a 1:1 ratio. This ensured individual-level age comparability and equal group sizes.

**Table S15.** Comparison of WMH volumes between symptomatic mutation carriers and age-matched controls using 1:1 nearest-neighbor matching.

| Mutation | Symptomatic group | | Age-matched control | | Wilcoxon W | p-value | Effect size |
| --- | --- | --- | --- | --- | --- | --- | --- |
|  | N | Age (Mean 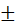 SD) | N | Age (Mean 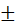 SD) |  |  |  |
| *C9orf72* | 77 | 64.99 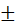 7.59 | 77 | 63.75 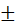6.9 | 3081 | 0.675 | 0.039 |
| *GRN* | 45 | 63.83 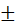 8.4 | 45 | 63.53 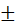 8.04 | 1275 | **0.034** | 0.259 |
| *MAPT* | 27 | 57.12 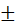 9.64 | 27 | 57.06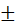 9.61 | 359 | 0.932 | 0.015 |

In the second approach, we applied a broader group-level match by selecting control participants whose ages fell within one standard deviation of the mean age of the symptomatic group for each mutation type. This strategy preserved more symptomatic cases while achieving similar age distributions at the group level.

**Table S16.** Comparison of WMH volumes between symptomatic mutation carriers and a group-level age-matched control sample (within ±1 SD of symptomatic group mean age).

| Mutation | Symptomatic group | | Age-matched control | | Wilcoxon W | p-value | Effect size |
| --- | --- | --- | --- | --- | --- | --- | --- |
|  | N | Age (Mean 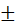 SD) | N | Age (Mean 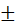 SD) |  |  |  |
| *C9orf72* | 77 | 64.99 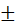 7.59 | 64 | 64.2 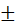 4.09 | 2542 | 0.748 | 0.032 |
| *GRN* | 45 | 63.83 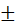 8.4 | 80 | 62.67 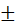 4.78 | 2387 | **0.002** | 0.326 |
| *MAPT* | 27 | 57.12 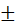 9.64 | 105 | 56.56 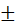 5.67 | 1755 | 0.057 | 0.238 |

In both analyses, WMH volumes were compared between symptomatic carriers and age-matched controls using Wilcoxon rank-sum tests. Effect sizes were calculated using rank-biserial correlations. These analyses confirmed that symptomatic GRN carriers consistently exhibited significantly higher WMH volumes, even when controlling for age differences, supporting the robustness of the main findings.

## GENFI consortium members

Annabel Nelson Department of Neurodegenerative Disease, Dementia Research Centre, UCL Queen Square Institute of Neurology, London, UK; Martina Bocchetta Department of Neurodegenerative Disease, Dementia Research Centre, UCL Queen Square Institute of Neurology, London, UK; David Cash Department of Neurodegenerative Disease, Dementia Research Centre, UCL Queen Square Institute of Neurology, London, UK; David L Thomas Neuroimaging Analysis Centre, Department of Brain Repair and Rehabilitation, UCL Institute of Neurology, Queen Square, London, UK; Emily Todd Department of Neurodegenerative Disease, Dementia Research Centre, UCL Queen Square Institute of Neurology, London, UK; Hanya Benotmane UK Dementia Research Institute at University College London, UCL Queen Square Institute of Neurology, London, UK; Jennifer Nicholas Department of Medical Statistics, London School of Hygiene and Tropical Medicine, London, UK; Kiran Samra Department of Neurodegenerative Disease, Dementia Research Centre, UCL Queen Square Institute of Neurology, London, UK; Rachelle Shafei Department of Neurodegenerative Disease, Dementia Research Centre, UCL Queen Square Institute of Neurology, London, UK; Carolyn Timberlake Department of Clinical Neurosciences, University of Cambridge, Cambridge, UK; Thomas Cope Department of Clinical Neuroscience, University of Cambridge, Cambridge, UK; Timothy Rittman Department of Clinical Neurosciences, University of Cambridge, Cambridge, UK; Antonella Alberici Centre for Neurodegenerative Disorders, University of Brescia, Brescia, Italy; Enrico Premi Stroke Unit, ASST Brescia Hospital, Brescia, Italy; Roberto Gasparotti Neuroradiology Unit, University of Brescia, Brescia, Italy; Valentina Cantoni Centre for Neurodegenerative Disorders, Department of Clinical and Experimental Sciences, University of Brescia, Brescia, Italy; Emanuele Buratti ICGEB, Trieste, Italy; Andrea Arighi Fondazione IRCCS Ca’ Granda Ospedale Maggiore Policlinico, Neurodegenerative Diseases Unit, Milan, Italy; University of Milan, Centro Dino Ferrari, Milan, Italy; Chiara Fenoglio Fondazione IRCCS Ca’ Granda Ospedale Maggiore Policlinico, Neurodegenerative Diseases Unit, Milan, Italy; University of Milan, Centro Dino Ferrari, Milan, Italy; Elio Scarpini Fondazione IRCCS Ca’ Granda Ospedale Maggiore Policlinico, Neurodegenerative Diseases Unit, Milan, Italy; University of Milan, Centro Dino Ferrari, Milan, Italy; Giorgio Fumagalli Fondazione IRCCS Ca’ Granda Ospedale Maggiore Policlinico, Neurodegenerative Diseases Unit, Milan, Italy; University of Milan, Centro Dino Ferrari, Milan, Italy; Vittoria Borracci Fondazione IRCCS Ca’ Granda Ospedale Maggiore Policlinico, Neurodegenerative Diseases Unit, Milan, Italy; University of Milan, Centro Dino Ferrari, Milan, Italy; Giacomina Rossi Fondazione IRCCS Istituto Neurologico Carlo Besta, Milano, Italy; Giorgio Giaccone Fondazione IRCCS Istituto Neurologico Carlo Besta, Milano, Italy; Giuseppe Di Fede Fondazione IRCCS Istituto Neurologico Carlo Besta, Milano, Italy; Paola Caroppo Fondazione IRCCS Istituto Neurologico Carlo Besta, Milano, Italy; Pietro Tiraboschi Fondazione IRCCS Istituto Neurologico Carlo Besta, Milano, Italy; Sara Prioni Fondazione IRCCS Istituto Neurologico Carlo Besta, Milano, Italy; Veronica Redaelli Fondazione IRCCS Istituto Neurologico Carlo Besta, Milano, Italy; David Tang-Wai The University Health Network, Krembil Research Institute, Toronto, Canada; Ekaterina Rogaeva Tanz Centre for Research in Neurodegenerative Diseases, University of Toronto, Toronto, Canada; Miguel Castelo-Branco Faculty of Medicine, University of Coimbra, Coimbra, Portugal; Morris Freedman Baycrest Health Sciences, Rotman Research Institute, University of Toronto, Toronto, Canada; Ron Keren The University Health Network, Toronto Rehabilitation Institute, Toronto, Canada; Sandra Black Sunnybrook Health Sciences Centre, Sunnybrook Research Institute, University of Toronto, Toronto, Canada; Sara Mitchell Sunnybrook Health Sciences Centre, Sunnybrook Research Institute, University of Toronto, Toronto, Canada; Christen Shoesmith Department of Clinical Neurological Sciences, University of Western Ontario, London, Ontario, Canada; Robart Bartha Department of Medical Biophysics, The University of Western Ontario, London, Ontario, Canada; Centre for Functional and Metabolic Mapping, Robarts Research Institute, The University of Western Ontario, London, Ontario, Canada; Rosa Rademakers Center for Molecular Neurology, University of Antwerp Jackie Poos Department of Neurology, Erasmus Medical Center, Rotterdam, Netherlands; Janne M. Papma Department of Neurology, Erasmus Medical Center, Rotterdam, Netherlands; Lucia Giannini Department of Neurology, Erasmus Medical Center, Rotterdam, Netherlands; Rick van Minkelen Department of Clinical Genetics, Erasmus Medical Center, Rotterdam, Netherlands; Yolande Pijnenburg Amsterdam University Medical Centre, Amsterdam VUmc, Amsterdam, Netherlands; Camilla Ferrari Department of Neuroscience, Psychology, Drug Research and Child Health, University of Florence, Florence, Italy; Enrico Fainardi Neuroradiology Unit, Department of Experimental and Clinical Biomedical Sciences, University of Florence, Florence, Italy; Stefano Chiti Neuroradiology Unit, Department of Experimental and Clinical Biomedical Sciences, University of Florence, Florence, Italy; Giulia Giacomucci Department of Neuroscience, Psychology, Drug Research and Child Health, University of Florence, Florence, Italy; Valentina Moschini Neurology unit , Careggi university Hospital, Florence Italy; Valentina Bessi Department of Neuroscience, Psychology, Drug Research and Child Health, University of Florence, Florence, Italy; Michele Veldsman Nuffield Department of Clinical Neurosciences, Medical Sciences Division, University of Oxford, Oxford, UK; Christin Andersson Department of Clinical Neuroscience, Karolinska Institutet, Stockholm, Sweden; Hakan Thonberg Center for Alzheimer Research, Division of Neurogeriatrics, Karolinska Institutet, Stockholm, Sweden; Linn Öijerstedt Center for Alzheimer Research, Division of Neurogeriatrics, Department of Neurobiology, Care Sciences and Society, Bioclinicum, Karolinska Institutet, Solna, Sweden; Unit for Hereditary Dementias, Theme Aging, Karolinska University Hospital, Solna, Sweden; Vesna Jelic Division of Clinical Geriatrics, Karolinska Institutet, Stockholm, Sweden; Paul Thompson Division of Neuroscience and Experimental Psychology, Wolfson Molecular Imaging Centre, University of Manchester, Manchester, UK; Tobias Langheinrich Division of Neuroscience and Experimental Psychology, Wolfson Molecular Imaging Centre, University of Manchester, Manchester, UK; Manchester Centre for Clinical Neurosciences, Department of Neurology, Salford Royal NHS Foundation Trust, Manchester, UK; Albert Lladó Alzheimer’s disease and Other Cognitive Disorders Unit, Neurology Service, Hospital Clínic, Barcelona, Spain; Anna Antonell Alzheimer’s disease and Other Cognitive Disorders Unit, Neurology Service, Hospital Clínic, Barcelona, Spain; Jaume Olives Alzheimer’s disease and Other Cognitive Disorders Unit, Neurology Service, Hospital Clínic, Barcelona, Spain; Mircea Balasa Alzheimer’s disease and Other Cognitive Disorders Unit, Neurology Service, Hospital Clínic, Barcelona, Spain; Nuria Bargalló Imaging Diagnostic Center, Hospital Clínic, Barcelona, Spain; Sergi Borrego-Ecija Alzheimer’s disease and Other Cognitive Disorders Unit, Neurology Service, Hospital Clínic, Barcelona, Spain; Ana Verdelho Department of Neurosciences and Mental Health, Centro Hospitalar Lisboa Norte - Hospital de Santa Maria Neuroscience Area, Biodonostia Health Research Insitute, San Sebastian, Gipuzkoa, Spain; Ana Gorostidi Neuroscience Area, Biodonostia Health Research Insitute, San Sebastian, Gipuzkoa, Spain; Jorge Villanua OSATEK, University of Donostia, San Sebastian, Gipuzkoa, Spain; Marta Cañada CITA Alzheimer, San Sebastian, Gipuzkoa, Spain; Mikel Tainta Neuroscience Area, Biodonostia Health Research Insitute, San Sebastian, Gipuzkoa, Spain; Miren Zulaica Neuroscience Area, Biodonostia Health Research Insitute, San Sebastian, Gipuzkoa, Spain; Myriam Barandiaran Cognitive Disorders Unit, Department of Neurology, Donostia University Hospital, San Sebastian, Gipuzkoa, Spain; Neuroscience Area, Biodonostia Health Research Insitute, San Sebastian, Gipuzkoa, Spain; Patricia Alves Neuroscience Area, Biodonostia Health Research Insitute, San Sebastian, Gipuzkoa, Spain; Department of Educational Psychology and Psychobiology, Faculty of Education, International University of La Rioja, Logroño, Spain; Benjamin Bender Department of Diagnostic and Interventional Neuroradiology, University of Tübingen, Tübingen, Germany; Lisa Graf Department of Neurodegenerative Diseases, Hertie-Institute for Clinical Brain Research and Center of Neurology, University of Tübingen, Tübingen, Germany; Annick Vogels Department of Human Genetics, KU Leuven, Leuven, Belgium; Mathieu Vandenbulcke Geriatric Psychiatry Service, University Hospitals Leuven, Belgium; Neuropsychiatry, Department of Neurosciences, KU Leuven, Leuven, Belgium; Philip Van Damme Neurology Service, University Hospitals Leuven, Belgium; Laboratory for Neurobiology, VIB-KU Leuven Centre for Brain Research, Leuven, Belgium; Rose Bruffaerts Department of Biomedical Sciences, University of Antwerp, Antwerp, Belgium; Biomedical Research Institute, Hasselt University, 3500 Hasselt, Belgium; Koen Poesen Laboratory for Molecular Neurobiomarker Research, KU Leuven, Leuven, Belgium; Pedro Rosa-Neto Translational Neuroimaging Laboratory, McGill Centre for Studies in Aging, McGill University, Montreal, Québec, Canada; Serge Gauthier Alzheimer Disease Research Unit, McGill Centre for Studies in Aging, Department of Neurology Reference Network for Rare Neurological Diseases (ERN-RND)Anne Bertrand Sorbonne Université, Paris Brain Institute – Institut du Cerveau – ICM, Inserm U1127, CNRS UMR 7225, AP-HP - Hôpital Pitié-Salpêtrière, Paris, France; Inria, Aramis project-team, F-75013, Paris, France; Centre pour l’Acquisition et le Traitement des Images, Institut du Cerveau et la Moelle, Paris, France; Aurélie Funkiewiez Centre de référence des démences rares ou précoces, IM2A, Département de Neurologie, AP-HP - Hôpital Pitié-Salpêtrière, Paris, France; Sorbonne Université, Paris Brain Institute – Institut du Cerveau – ICM, Inserm U1127, CNRS UMR 7225, AP-HP - Hôpital Pitié-Salpêtrière, Paris, France; Daisy Rinaldi Centre de référence des démences rares ou précoces, IM2A, Département de Neurologie, AP-HP - Hôpital Pitié-Salpêtrière, Paris, France; Sorbonne Université, Paris Brain Institute – Institut du Cerveau – ICM, Inserm U1127, CNRS UMR 7225, AP-HP - Hôpital Pitié-Salpêtrière, Paris, France; Département de Neurologie, AP-HP - Hôpital Pitié-Salpêtrière, Paris, France; Dario Saracino Sorbonne Université, Paris Brain Institute – Institut du Cerveau – ICM, Inserm U1127, CNRS UMR 7225, AP-HP - Hôpital Pitié-Salpêtrière, Paris, France; Inria, Aramis project-team, F-75013, Paris, France; Centre de référence des démences rares ou précoces, IM2A, Département de Neurologie, AP-HP - Hôpital Pitié-Salpêtrière, Paris, France; Olivier Colliot Sorbonne Université, Paris Brain Institute – Institut du Cerveau – ICM, Inserm U1127, CNRS UMR 7225, AP-HP - Hôpital Pitié-Salpêtrière, Paris, France; Inria, Aramis project team, F-75013, Paris, France; Centre pour l’Acquisition et le Traitement des Images, Institut du Cerveau et la Moelle, Paris, France; Sabrina Sayah Sorbonne Université, Paris Brain Institute – Institut du Cerveau – ICM, Inserm U1127, CNRS UMR 7225, AP-HP - Hôpital Pitié-Salpêtrière, Paris, France; Catharina Prix Neurologische Klinik, Ludwig-Maximilians-Universität München, Munich, Germany; Elisabeth Wlasich Neurologische Klinik, Ludwig-Maximilians-Universität München, Munich, Germany; Olivia Wagemann Neurologische Klinik, Ludwig-Maximilians-Universität München, Munich, Germany; Sandra Loosli Neurologische Klinik, Ludwig-Maximilians Universität München, Munich, Germany; Sonja Schönecker Neurologische Klinik, Ludwig-Maximilians-Universität München, Munich, Germany; Tobias Hoegen Neurologische Klinik, Ludwig-Maximilians-Universität München, Munich, Germany; Jolina Lombardi Department of Neurology, University of Ulm, Ulm; Sarah Anderl-Straub Department of Neurology, University of Ulm, Ulm, Germany; Adeline Rollin CHU, CNR-MAJ, Labex Distalz, LiCEND Lille, France; Gregory Kuchcinski Univ Lille, France; Inserm 1172, Lille, France; CHU, CNR-MAJ, Labex Distalz, LiCEND Lille, France; Maxime Bertoux Inserm 1172, Lille, France; CHU, CNR-MAJ, Labex Distalz, LiCEND Lille, France; Thibaud Lebouvier Univ Lille, France; Inserm 1172, Lille, France; CHU, CNR-MAJ, Labex Distalz, LiCEND Lille, France; Vincent Deramecourt Univ Lille, France; Inserm 1172, Lille, France; CHU, CNR-MAJ, Labex Distalz, LiCEND Lille, France; Beatriz Santiago Neurology Department, Centro Hospitalar e Universitario de Coimbra, Coimbra, Portugal; Diana Duro Faculty of Medicine, University of Coimbra, Coimbra, Portugal; Maria João Leitão Centre of Neurosciences and Cell Biology, Universidade de Coimbra, Coimbra, Portugal; Maria Rosario Almeida Faculty of Medicine, University of Coimbra, Coimbra, Portugal; Miguel Tábuas-Pereira Neurology Department, Centro Hospitalar e Universitario de Coimbra, Coimbra, Portugal; Sónia Afonso Instituto Ciencias Nucleares Aplicadas a Saude, Universidade de Coimbra, Coimbra, Portugal
